# The role of the Hes1 crosstalk hub in Notch-Wnt interactions of the intestinal crypt

**DOI:** 10.1371/journal.pcbi.1005400

**Published:** 2017-02-28

**Authors:** Sophie K. Kay, Heather A. Harrington, Sarah Shepherd, Keith Brennan, Trevor Dale, James M. Osborne, David J. Gavaghan, Helen M. Byrne

**Affiliations:** 1 Department of Computer Science, University of Oxford, Oxford, U.K.; 2 Mathematical Institute, University of Oxford, Oxford, U.K.; 3 School of Mathematical Sciences, University of Nottingham, Nottingham, U.K.; 4 Wellcome Trust Centre for Cell Matrix Research, University of Manchester, Manchester, U.K.; 5 School of Biosciences, Cardiff University, Cardiff, U.K.; 6 School of Mathematics and Statistics, University of Melbourne, Melbourne, Australia; University of Michigan Medical School, UNITED STATES

## Abstract

The Notch pathway plays a vital role in determining whether cells in the intestinal epithelium adopt a secretory or an absorptive phenotype. Cell fate specification is coordinated via Notch’s interaction with the canonical Wnt pathway. Here, we propose a new mathematical model of the Notch and Wnt pathways, in which the Hes1 promoter acts as a hub for pathway crosstalk. Computational simulations of the model can assist in understanding how healthy intestinal tissue is maintained, and predict the likely consequences of biochemical knockouts upon cell fate selection processes. Chemical reaction network theory (CRNT) is a powerful, generalised framework which assesses the capacity of our model for monostability or multistability, by analysing properties of the underlying network structure without recourse to specific parameter values or functional forms for reaction rates. CRNT highlights the role of *β*-catenin in stabilising the Notch pathway and damping oscillations, demonstrating that Wnt-mediated actions on the Hes1 promoter can induce dynamic transitions in the Notch system, from multistability to monostability. Time-dependent model simulations of cell pairs reveal the stabilising influence of Wnt upon the Notch pathway, in which *β*-catenin- and Dsh-mediated action on the Hes1 promoter are key in shaping the subcellular dynamics. Where Notch-mediated transcription of Hes1 dominates, there is Notch oscillation and maintenance of fate flexibility; Wnt-mediated transcription of Hes1 favours bistability akin to cell fate selection. Cells could therefore regulate the proportion of Wnt- and Notch-mediated control of the Hes1 promoter to coordinate the timing of cell fate selection as they migrate through the intestinal epithelium and are subject to reduced Wnt stimuli. Furthermore, mutant cells characterised by hyperstimulation of the Wnt pathway may, through coupling with Notch, invert cell fate in neighbouring healthy cells, enabling an aberrant cell to maintain its neighbours in mitotically active states.

## Author summary

Epithelial cells which line the intestine form finger-shaped structures called crypts; these undergo a process of renewal at the base, causing cells to migrate upwards until they die and are sloughed off into the gut. Much of our understanding of how crypts function rests upon two processes: proliferation, in which cells divide to produce ‘daughter cells’; and differentiation, in which cells become progressively more specialised as they migrate along the crypt axis and mature. Coordinated proliferation and differentiation enable each crypt to renew itself and to produce a range of specialised cell types essential to its healthy functioning. In this paper we build a mathematical model for two reaction pathways which regulate proliferation and differentiation. We use this model to explore how crosstalk between these pathways in cell pairs influences the generation of distinct cell fates in intestinal tissues. By modifying our model to represent abnormal, ‘mutant’ cells, we investigate abnormalities typical of early colorectal cancer. Computational simulation of our model identifies an important region of crosstalk in our reaction network which determines whether cells adopt the same fate as one another, or different fates. Our model may prove useful for realistic simulations of whole crypts in the future.

## Introduction

**Fig 1:**
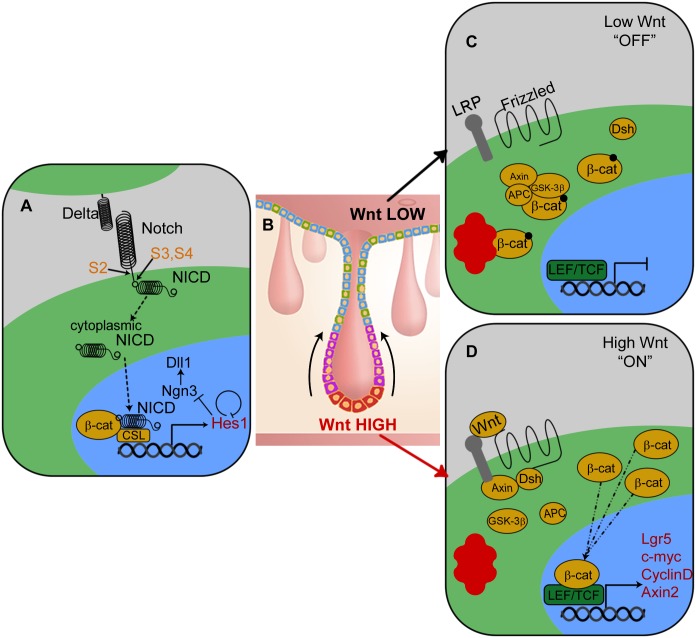
Biological models for cell signalling in the intestinal crypt epithelium. Simplified schematics depict: (A) the Notch pathway, where labels S2-S4 indicate cleavage sites in the Notch receptor; (B) a cross-section of a crypt in the large intestine. Arrows denote the general direction of cell migration. Cells are coloured according to their type: red indicates a stem cell; purple indicates a proliferative progenitor or transit cell; secretory goblet cells are shown in green, and absorptive enterocytes are shown in blue; (C) the Wnt pathway in its low-stimulus state; (D) the Wnt pathway in its high-stimulus state. All network diagrams depict the cytoplasm in green, the nucleus in blue, the intercellular space in grey and the proteasome in red, while transcriptional targets of the pathway are listed in red. Image (B) is adapted from Reizel *et al*. [8], originally published by PLOS and provided under a Creative Commons Attribution Licence, *CC-BY-2.5*.

Attainment and maintenance of homeostasis within the epithelial lining of the intestine is achieved through a nuanced coordination of biochemical processes and spatial cues within the tissue, in particular those which influence proliferation and cell fate selection. Crosstalk between the subcellular pathways governing these processes facilitates the coordination of cellular division and specialisation throughout the tissue of the intestinal epithelium, producing the broad range of cell types required for its function, ranging from totipotent stem cells, to terminally differentiated secretory or absorptive phenotypes. These cells collectively form test-tube shaped structures called *crypts*, each consisting of up to 700 cells in mice [1] and 2000 in humans [2], with millions of such crypts distributed throughout the intestine. As shown in the schematic of Fig 1, cells proliferate in the lower regions of the crypt [3] and (with the exception of specialised Paneth cells [4]) migrate upwards, until they die and are sloughed into the lumen of the gut [5]. Increased specialisation occurs as cells migrate up the crypt, arguably due to spatial cues in the surrounding tissue [6, 7]. Consequently the interlacing of proliferative and differential processes is key to understanding the attainment and maintenance of homeostasis in the healthy intestinal epithelium, or indeed how this is perturbed in conditions such as colorectal cancer.

**Fig 2:**
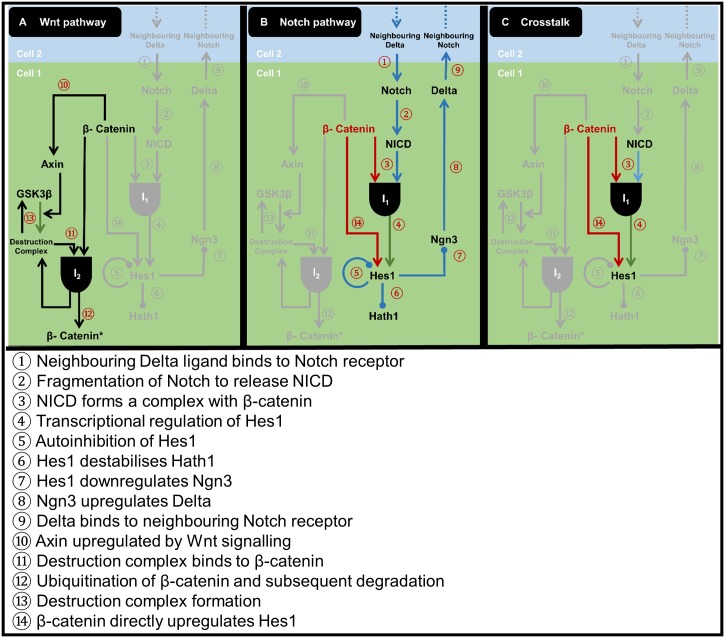
Network representation for our model of Notch-Wnt interaction. Our model comprises: (A) the Wnt pathway, (B) the Notch pathway, and (C) crosstalk points. Numbered steps are justified in S1 Text. Major steps involving the *β*-catenin crosstalk hub are shown in red, while Wnt-dependent steps are shown in purple. Black AND gates signify the formation of intermediate complexes from two molecular partners. Circular end caps indicate inhibition steps.

In this paper we focus on interactions between the canonical Wnt pathway, in its role of mitotic regulator, and the Notch pathway, in its role of cell fate specifier. Both pathways facilitate responses to spatial cues within the tissue: the canonical Wnt pathway characterises the cellular response to local extracellular concentrations of Wnt, whilst the Notch pathway coordinates cell fate progression in populations of neighbouring cells. Schematics of the biochemical models for each pathway are shown in Fig 2.

The *Wnt pathway* is crucial in the development and maintenance of biological tissues. Its canonical form centres on a mechanism for the control of cytoplasmic levels of *β*-catenin, which assists in regulating transitions through the cell cycle. A key element in the regulation of cytoplasmic *β*-catenin levels is a ‘destruction complex’. This is an aggregate of scaffold proteins including GSK [9], Axin [10, 11] and APC [12, 13] that can phosphorylate *β*-catenin, labelling it for ubiquitination and subsequent destruction by the proteasome. When the extracellular Wnt stimulus is high, Wnt proteins bind to Frizzled receptors at the cell surface membrane and recruit cytoplasmic Dishevelled (*Dsh*) to the cell membrane [14]. *Dsh* inhibits the destruction complex, thus reducing the phosphorylation and degradation of *β*-catenin. There is an increase in cytoplasmic levels of *β*-catenin and, consequently, increased translocation of *β*-catenin to the nucleus, where it binds to LEF/TCF proteins and induces production of associated target genes, including some associated with cell-cycle progression [15]. *β*-catenin also transcriptionally upregulates the functionally-conserved Axin homologue, Axin2 [10, 16], forming a negative feedback loop to regulate the Wnt pathway. When the extracellular Wnt stimulus is low, Frizzled receptors are largely unbound and Dishevelled is inactive. The destruction complex forms and binds to cytoplasmic *β*-catenin. Once *β*-catenin is bound to the destruction complex, it is phosphorylated [13], ubiquitinated and destroyed by the proteasome [17]. Translocation of *β*-catenin to the nucleus is limited and the production of target genes is reduced.

The canonical Wnt pathway is therefore constitutively active in cells exposed to high levels of the protein Wnt, and inactive when the local Wnt concentration is low. There are many different forms of Wnt, with nineteen distinct Wnt genes known to exist in the mammalian genome [18]. The strength of the extracellular Wnt signal is a key factor in Wnt pathway activity; we note that the intestinal crypt exhibits a spatial variation in extracellular Wnt along the crypt axis, with a high concentration at the crypt base which tapers off towards the crypt mouth [19]. Non-canonical forms of Wnt signalling govern a variety of processes, including: integrin-mediated intercellular adhesion; planar cell polarity, and Wnt/calcium signalling [11]. In this study, we focus on the canonical pathway; discussion of other modes of Wnt action can be found in the reviews [20–23].

A number of mathematical models of the canonical Wnt pathway have been proposed, typically using ordinary differential equations (ODEs). Of particular note is the ODE model of Lee *et al*. [24], which proposes that Axin, a component of the *β*-catenin destruction complex, acts as a rate-limiter in *β*-catenin degradation and may provide a robust regulator for the strength of Wnt signalling within a cell. Model simulations [24, 25] have led to suggestions that Axin might regulate crosstalk between Wnt and other pathways (e.g. MAPK), by stabilising levels of other members of the destruction complex such as APC. Systematic analyses have yielded reduced ODE systems [26–28], have reformulated the model as a system of delay differential equations [29], have added inhibitory Wnt targets to generate oscillations [30, 31], or have extended the network to resolve the *β*-catenin degradation processes in greater detail [32] or to examine crosstalk with glycosylation pathways [33]. Computational evaluation [34] of the model of Lee *et al*. found that the fold-change in, rather than the absolute expression of, *β*-catenin was the most robust feature to parameter perturbations, given a fixed Wnt stimulus. This fold-change may act as a means of overcoming biological noise and is observed experimentally in human colorectal cell lines [34]. Recalibration of the model of Lee *et al*. for several human and canine cell lines [35] suggests that higher Axin and lower APC concentrations are required when applying the model in a mammalian context, rather than the *Xenopus* oocytes of Lee *et al*. Alternative mathematical models of the Wnt pathway adopt shuttling and/or compartmental approaches, accounting for subcellular localisation of proteins in the nucleus, cytoplasm and cell membrane [36–41].

The *Notch pathway* regulates the transition from fully undifferentiated to terminally differentiated cell and belongs to the class of *juxtacrine* signalling networks [42]. Juxtacrine signals are initiated by contact-based processes, in which two or more adjacent cells transfer signals via ligand binding events at their cell surface membranes [43]. In the intestinal epithelium, the Notch pathway is associated with the generation of alternating cellular patterns involving secretory and absorptive cell phenotypes.

The Notch pathway operates as follows: a membrane-bound Delta ligand on a signalling cell can bind with a Notch receptor on a neighbouring cell [44], initiating a series of reactions in the latter cell which constitute a *Notch signalling cascade* [42]. Delta-Notch binding induces three cleavage events in the Notch ligand at the cell membrane [45]; mediated in part by the enzyme complex, *γ*-secretase, these cleavages release a fragment known as the Notch Intra-Cellular Domain (NICD) into the cytoplasm [46]. NICD binds directly to *β*-catenin [47] and translocates to the nucleus, where it associates with the protein CSL to form a transcriptional activator complex [48] which induces transcription of target genes, in particular *Hes1*. The resulting *Hes1* protein dimerises and binds to its own promoter, thereby negatively regulating its own expression [49–51] and causing oscillations in the levels of both *Hes1* mRNA and protein [52]. In addition, *Hes1* either inhibits transcription of the proneural protein *Ngn3* [53] or destabilises it [54]. *Ngn3* would otherwise upregulate the Delta gene, *Dll*1 [54–57], and so the strength of the Notch signal is inversely linked with Delta expression [53, 58].

There are four types of differentiated cell in the epithelium of the small intestine: goblet, Paneth, and enteroendocrine, which are secretory; and enterocytes, which are absorptive [59, 60]. The Notch pathway plays an important role in selecting which cell fate will be realised, via the Notch target *Hes1*, and the protein *Hath1*, which is normally suppressed by *Hes1* [61–63]. Expression of *Hath1* (and suppression of *Hes1*) is associated with secretory phenotypes [58], whilst high *Hes1* expression correlates with an absorptive fate [62, 64]. *In vivo* studies on the small intestine of *Hes1*-knockout mice have generated substantial numbers of all three secretory cell types [53]. Similarly, inhibition of Notch signalling yields an increased population of goblet cells [46]. Gain- and loss-of-function studies by Fre *et al*. [62] support this theory and suggest that Notch signalling via *Hes1* is responsible for early-stage cell fate selection. As a cell migrates through the epithelium and is exposed to reduced Wnt levels, other pathways and cell regulators coordinate a more refined selection of fate: they may, for example, determine a specific fate for a cell which has already been selected for secretory function [60, 65]. Understanding how *Hes1* and *Hath1* expression levels are regulated within a cell therefore offers valuable insights into the cell fate selection process [66–68].

Many mathematical models of Notch signalling focus on receptor-ligand binding, using either discrete difference equations or continuous ODEs. Such models have captured lateral inhibition [69, 70] or lateral induction [71–73] and may be embedded in individual cells of a lattice to generate cell patterns. Other models distinguish between cis- and trans-Delta in the Notch binding events, in which Notch may bind to either a *cis-*Delta ligand on the same cell, or a *trans-*Delta ligand on a neighbouring cell; this generates strong switch-like behaviour [74]. Many mathematical models have included subcellular detail of the Notch pathway, typically building on the Goodwin model for negative feedback [75], which employs ordinary differential equations and augments a system of mRNA and protein with an unspecified intermediate in order to generate sustained oscillations, by creating a distributed delay in the system [76]. Hirata *et al*. [52] follow Goodwin and propose a simple ODE model which achieves an experimentally-validated, two hour oscillation period for *Hes1*. Other reformulations of the Goodwin framework highlight the functional importance of transcriptional delays in modelling oscillations in *Hes1* mRNA and protein levels [77, 78], with dimerisation of *Hes1* protein yielding oscillation amplitudes in closer agreement with experimental data [79]. Autorepression of *Hes1* has been shown to aid the tunability of oscillations when two *Hes1* oscillators are coupled [80]. Recent stochastic models of the Hes1 autoregulation network have emphasised the role of molecular compartmentalisation, in particular the location of Hes1 dimerisation, as an important factor in matching Hes1 expression to experimental data [81–84]. Moreover, the stochastic compartmental approach is sufficient to generate heterogeneity [82]. Detailed subcellular models of the Notch pathway suggest that variation in transcriptional repression of *Hes1* may facilitate the transition between oscillation and bistability [85] and may be used to generate early cell-fate selection [86], although crosstalk with another pathway is required for terminal differentiation [86].

Simple mathematical and computational models for Notch-Wnt interaction are also employed in multicellular settings to study the effects of spatial variation in Wnt stimuli upon cell fate selection. Some of these models eschew equation systems altogether, in favour of a rule-based paradigm in which Notch and Wnt activity are each either high or low and the system evolves according to an averaging process over neighbouring cells [87, 88]. However, this pared-down approach cannot represent subcellular details of the crosstalk. Existing mathematical models at the subcellular scale have accounted for interactions between GSK3*β* and NICD [89], between NICD and Dsh [90] or via LEF/TCF and membrane-bound Notch, in which inhibition of Wnt is used to drive terminal differentiation [91, 92]. Some of these models omit a *Hes1* autoregulation motif [91, 92] and indeed, few multicellular studies of the role of *Hes1* oscillations in cell fate exist at the present time. Oscillations of *Hes1* are thought to assist in the maintenance of cell-cycle processes and the flexibility of cell fate decisions within a cell population [93].

The dynamic modelling of intracellular signalling pathways described here can take a variety of forms: these include deterministic models, in which time-dependent (often continuous) equations define a system evolution towards specific future states; and stochastic models, in which probabilistic elements introduce randomised aspects to the evolution [94–97]. Stochastic models are of particular relevance when modelling situations involving small numbers of molecules, in which a continuum approximation may not be valid [98]. Many of the existing models for Notch (e.g. [69, 79, 85]) and Wnt (e.g. [24, 32]) adopt a continuous, deterministic approach; others have introduced stochastic expression of Hes1 protein [81–84] or *β*-catenin [99].

Our main focus here is the development of a mathematical model of deterministic ODEs for the interaction of the Notch and Wnt pathways in the cells of the intestinal epithelium. Given the rich complexity which reaction networks and their crosstalk generate, computational approaches form a cornerstone of the analysis and implementation of our model. In particular, chemical reaction network theory (CRNT) offers a powerful means of analysing the steady-state behaviour of our model without recourse to either specific parameter values or functional forms for the reaction dynamics. CRNT facilitates the analysis of large and complex networks and is performed with a stand-alone, ready-to-use computational toolbox [100]. Having derived, parametrised and calibrated our model, we use it to explore two-cell systems in healthy and malignant scenarios, an approach which has proved insightful in other mathematical studies of Notch regulation [101]. These cell pair settings demonstrate the main dynamic features of the model, illustrate the role of Notch-Wnt crosstalk in shaping the cell fate response, and permit preliminary investigation of the possible consequences of dysregulation of components of the two pathways in the subcellular network.

## Results

The primary aim of our mathematical model is to study how crosstalk between the Notch and Wnt pathways—in particular activity via the *Hes1* promoter—influences cell fate selection. A schematic of the biochemical network which our model represents is shown in Fig 2. Each of the 14 steps in our network model is supported by experimental evidence.

### Derivation of Notch-Wnt crosstalk model

The elements of our Wnt sub-model (five species) represent the shuttling of GSK3*β* between a non-complexed form (*G*) and a complexed form (*C*), and their effects upon *β*-catenin (*B*) and Axin levels (*A*) (Fig 2A). We refer to *C* as the ‘destruction complex’ (comprising GSK3*β*, Axin and—implicitly—APC) because of its role in targeting *β*-catenin for ubiquitination and degradation, although we do not explicitly represent these two processes in our model. Instead we consider a complex *I*_2_, which comprises the destruction complex bound to *β*-catenin and which degrades to release only *C* back into the system.

The components of our Notch submodel (seven species) encapsulate the binding of Delta ligand (*D*) and Notch receptor (*N*) at the cell surface membrane [44] and the resulting subcellular signalling cascade [42] responsible for *Hes1* regulation (Fig 2B). Given our focus on crosstalk with the Wnt pathway, our Notch submodel incorporates NICD (*F*) arising from Notch cleavage; an intermediate complex (*I*_1_) formed when NICD binds to *β*-catenin; the cell fate specifiers Hes1 (*H*_1_) and Hath1 (*H*_2_); and the proneural protein Ngn3 (*P*).

Our focus on Notch-Wnt interaction motivates us to consider the crosstalk hub involved with Hes1 regulation (Fig 2C). Here, Hes1 regulation is governed by two main routes. The *Notch-mediated* route (Steps ③ and ④) involves the binding of *I*_1_ to the promoter. This interaction has been identified in vascular progenitor cells [47] and human kidney cells [102] and may provide a switching mechanism in the canonical Wnt pathway by diverting *β*-catenin from regulating Wnt target genes [102]. In the *Wnt-mediated* route, regulation occurs via *β*-catenin binding alone (Step ⑭); this Notch-independent mechanism is supported by Peignon *et al*. [103], who have identified complementary binding sites on the *β*-catenin molecule and Hes1 promoter and infer direct regulation of Hes1 levels by *β*-catenin.

We also incorporate a Wnt-mediated intervention involving the downregulation of Hes1 by Dishevelled [48]. Experimental evidence [48] reveals a 0.4− to 0.5−fold change in Notch activity in response to expression of either Wnt or Dsh, yet expression of *β*-catenin results in a 1.2−fold change. This interaction is thought to occur through binding and reduction in the levels of a NICD coactivator, CSL, and is distinct from the direct, ‘Notch-mediated’ regulation mechanism described above. For simplicity, we do not model the concentration of Dsh, but we scale the production of Hes1 by a function of the local Wnt stimulus to simulate this effect.

The interactions which comprise our network model are based upon the following assumptions:

- Decay rates follow a first-order mass action law (species *X* decays at rate *μ*_*X*_ *X*, where *μ*_*X*_ represents the rate of decay);
- Binding reactions between *β*-catenin and NICD, and between *β*-catenin and the destruction complex, follow first-order mass action laws;
- The rate of upregulation events in the Notch system, and of Axin in the Wnt system, are modelled using Hill functions;
- Inhibition events are represented by hyperbolas and include the Dsh-mediated downregulation of Hes1, denoted by a Wnt-dependent function Ψ_*W*_;
- Incorporation of non-complexed GSK3*β* into the destruction complex (*G* → *C*) is represented by a function Ψ_*W*,*A*_, which depends on the subcellular Axin concentration *A* and the extracellular Wnt concentration *W*; the reverse reaction (*C* → *G*) is assumed to occur at a constant rate.

Under these assumptions, we realise the reaction network as a system of twelve ordinary differential equations (ODEs), shown in Eqns. (S.1)–(S.13) of S1 Text. These take the form $\dot{x}=Y\left(x,k\right)$, where **x** is a vector of the twelve network species, $\dot{x}$ represents the time derivative of the species concentrations, and the vector **k** contains the model parameters (see Tables S.3–S.6 of S1 Text).

### Steady-state analysis

The steady state behaviour of the mathematical model is used to confirm qualitative matching with known biological features. Given the size of the system (12 variables and 41 parameters, Table S.2 of S1 Text), this analysis is restricted to a ‘single cell’ scenario, commensurate with a homogeneous population of cells, in which the Notch and Wnt subnetworks decouple. Terms for NICD in the Wnt system, or *β*-catenin in the Notch system, are treated as input parameters rather than variables. This renders tractable analysis of each subnetwork.

The Wnt submodel (Fig 2A) yields readily to mathematical analysis and it is straightforward to derive an implicit expression for the steady state of *β*-catenin (B*), along with explicit expressions for the other species. B* exhibits qualitatively appropriate behaviour within the Wnt system. For instance, B* is increased by: increasing the Wnt stimulus; increasing the production rate of *β*-catenin; decreasing the rate constant for formation of the destruction complex; or by decreasing the rate at which the destruction complex binds to *β*-catenin. Steady state analysis also suggests that strong interaction of *β*-catenin with NICD attenuates the response of *β*-catenin to variation in Wnt levels. When the crosstalk with Notch is reduced, B* increases with the level of the Wnt signal.

Steady-state analysis of our Notch submodel (Fig 2B) is more complex. In the absence of suitable quantitative data, we make a simplifying assumption based upon the structure of our Notch model to facilitate its analysis: we assume that the concentrations of NICD and the complex of NICD bound to *β*-catenin are linearly proportional to that of Notch. Under this assumption, the model simplifies to four equations (for Notch, Hes1, proneural protein and Delta). The ensuing analysis demonstrates the existence of a steady state for Hes1, and hence for the other elements of the pathway. Linear stability analysis identifies oscillatory dynamics for Hes1, associated with the parameter *θ*_2_, which governs the balance of Wnt-mediated and Notch-mediated transcription of Hes1. In particular, our analysis suggests that the action of *β*-catenin upon the Hes1 promoter stabilises Notch and dampens oscillations. This agrees with an earlier mathematical model of Hes1 regulation [85] in which the transcriptional repression of Hes1 was varied to achieve a transition between oscillation and bistable switching.

Our studies of the decoupled Notch and Wnt systems confirm the existence of biologically realistic steady states and, in the case of Notch, regimes which yield damped oscillations. These findings are qualitatively consistent with experimentally observed behaviour for oscillations in the Notch system [52, 78] as well as existing mathematical representations of oscillation [69, 77, 85]. Similarly the *β*-catenin response in the Wnt system detailed here is consistent with qualitative behaviour reported in the experimental literature [104].

### Transcriptional regulation of Hes1 is central to dynamics of the decoupled system

Traditional steady state analysis, in the sense of the pure analytic approach described above, is impeded by the complexity of the reaction network. Insights into the influence of crosstalk require alternative methods capable of analysing the full network. For example, we would like to establish whether the full model exhibits multiple steady states. Often one would like to preclude or assert particular dynamic behaviour, even if model parameters change from their estimated values, or if the functional form of terms in the model changes. *Chemical reaction network theory* (CRNT) [105] can determine the multistationarity properties of the network without specifying either parameter values or explicit functional forms for the dynamics and without recourse to the system reductions employed in the Notch steady-state analysis described above.

CRNT identifies how coupling of the subnetworks affects the stability of the system, and achieves this via testable properties derived from the network structure. Specifically, we want to know whether a network is *concordant*, a property which relates to its physical architecture in a parameter- and equation-free setting. A full definition of this property is technically involved; further details are provided in Methods and Models and a complete mathematical description in S1 Text (including some examples of concordant networks in Fig. S.3 of S1 Text). The *Chemical Reaction Network Toolbox* [100] provides purpose-built network analysis algorithms which can assess our network—or its constituent subnetworks—for concordance.

Shinar and Feinberg [105] proved that when a network is *weakly monotonic* (i.e. increasing the rate of a particular reaction increases the concentration of at least one of its reactant species) and *concordant*, then multiple steady states are precluded, as are degenerate positive steady states. For networks satisfying additional properties (*weakly reversible*, *conservative*, continuous kinetics, and non-zero initial condition), there will be precisely one steady state, for which all species concentrations are strictly positive. In some cases (smooth kinetics in which each species has an associated degradation), concordance can assert that the unique steady state is stable, in that every real eigenvalue associated with it has negative real part. CRNT can therefore indicate the general stability properties of our reaction system by determining concordance, or lack thereof, and a broad classification for the functional forms used to describe the reaction rates of the network; it does not require a specific instantiation of the model.

Results from CRNT analysis of the decoupled and full systems of our model are given in Table 1. Inspection of our model equations (S.1)–(S.13) of S1 Text confirms that our model satisfies the requirements for weakly monotonic kinetics allied with an influence specification (which describes the species which up- or down-regulate each reaction) and so the results of Shinar and Feinberg apply. Concordant subnetworks within our model are therefore monostable, whilst discordant subnetworks are multistable [105].

**Table 1:** Chemical reaction network results for decoupled and full system. Concordance results for decoupled and whole networks in a homogeneous system, analysed using the CRN Toolbox [100]. A tick indicates a concordant network (monostable) and a cross, a discordant network (multistable). The second column indicates which *β*-catenin crosstalk points were included in each network. *β*-catenin is not consumed in Step 14 and so the comparison of the two coupling points does not apply to the Wnt-only system.

Model	Active Coupling Points	Wnt ON	Wnt OFF
*Notch Only*	Step ④ only Step ④ & Step ⑭	✔ ✔	✘ ✔
*Wnt Only*	N/A	✔	✔
*Full System*	Step ④ only Step ④ & Step ⑭	✘ ✔	✘ ✔

#### Discordance of the Notch subnetwork influences the whole system

CRNT’s principal finding is in identifying the Notch subnetwork as discordant, and hence multistable; furthermore, this discordance carries through to the coupled Notch-Wnt network. Comparisons between the decoupled and full systems yield three valuable insights into the origins of the behaviour of the coupled network:

1. ***β*-catenin acts as a stabilising force:** Direct action of *β*-catenin upon the Hes1 promoter (networks involving Step ⑭) always yields an injective, and hence monostable, network. *β*-catenin serves to dampen the multistability associated with the Notch system. Conversely, all discordant networks in this analysis arise when Hes1 transcription is solely Notch-mediated.
2. **Discordance arising from Wnt response:** In the presence of a Wnt stimulus, monostable Notch and Wnt subnetworks can combine to produce a multistable network. This occurs when Hes1 transcription is solely Notch-mediated. Harrington *et al*. [106] demonstrate that in such cases, the rate of shuttling of crosstalk species (in our case, *β*-catenin) between the subnetworks determines the region of parameter space in which multistability occurs.
3. **Multistability in Notch influences the full system:** Sub-networks can transmit their discordance to the full network, as shown by Joshi and Shiu [107]. Some of the discordance in the full system (Step ④ only, Wnt off) can be attributed to the equivalent discordance in the Notch system.

The results in Table 1 show that the stability properties of the full system depend not just on the presence or absence of a Wnt stimulus, but also on the mechanisms governing transcription of Hes1, Steps ④ and ⑭ of the model. In particular, the relative contribution of Notch- and Wnt-mediated transcription of Hes1 determines whether the full system is monostable or multistable. Biologically this corresponds to Notch favouring heterogeneity and flexibility for cell fate selection, whereas Wnt influences the system towards a single steady state.

In light of these findings, the subsequent two-cell studies focus upon the relative contribution of Notch- and Wnt-mediated regulation of Hes1 and the strength of the Wnt stimulus *W*, with a view to understanding how activity around the Hes1 crosstalk hub delivers the coordination of fate selection seen in tissues of the intestinal epithelium.

### Implications of cross-talk for heterogeneous states

The next application focuses upon *in silico* studies of a healthy cell pair using the complete network (both Notch and Wnt pathways, and their crosstalk) shown in Fig 2A–2C, in which the governing parameters for Hes1 transcription can be manipulated to deliver either Wnt-dominant or Notch-dominant control of the Hes1 promoter. Since cell populations in tissues are naturally heterogeneous, we focus on *two coupled heterogeneous cells*, each running an embedded system of the twelve model ODEs, using the parametrisation described in Tables S.3–S.7 of S1 Text. Coupling between cells is achieved by identifying the Delta value of a particular cell as the “mean neighbouring Delta” component of the Notch equation in its neighbour, as shown in Eqn. (S.1) of S1 Text. Both cells start from the standard Wnt conditions listed in Table S.7 of S1 Text; the first cell of the pair adopts conditions of 0.5*nM* for all its Notch components, whilst the Notch entities of the second cell start from 0.51*nM*. This difference permits the emergence of heterogeneous states.

**Fig 3:**
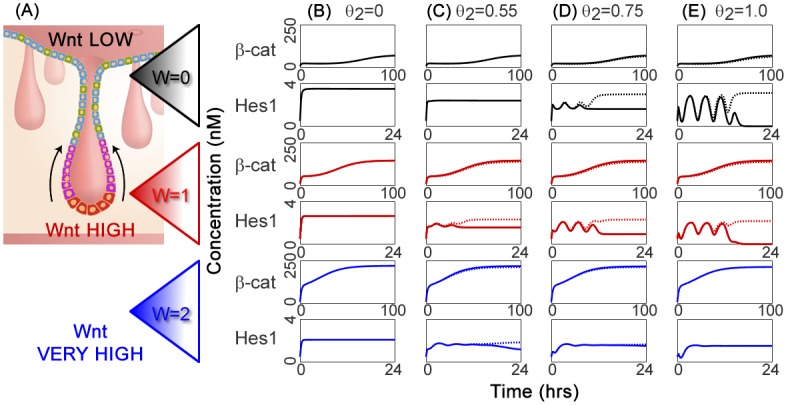
Plots showing how variation in *θ*_2_, the proportion of Notch-mediated control of the Hes1 promoter, affects the system dynamics. (A) Cross-section schematic of a crypt from the large intestine; image adapted from Reizel *et al*. [8], originally published by PLoS and provided under a Creative Commons Attribution Licence, *CC-BY-2.5*. (B–E) Influence of *θ*_2_ upon the Hes1 steady state at *W* = 0.0 (black), *W* = 1.0 (red) and *W* = 2.0 (blue); *θ*_2_ ∈ [0, 1] represents the proportion of Notch-mediated transcription of Hes1. Timecourses show Hes1 and *β*-catenin expression for healthy cell pairs, for (B) *θ*_2_ = 0.00 (i.e. entirely Wnt-mediated), (C) *θ*_2_ = 0.55, (D) *θ*_2_ = 0.75, (E) *θ*_2_ = 1.00 (i.e. entirely Notch-mediated). The timecourse for the first cell of each pair is indicated by a solid line; the second, by a dotted line. Where only one timecourse is apparent, the cell pair is synchronised. Standard initial conditions and parameters are used, stated in Tables S.3–S.7 of S1 Text; the ODE model comprises equations (S.1)–(S.13) of S1 Text.

Simulation results from these healthy cell pairs are displayed in Fig 3B–3E. In our model, the parameter *θ*_2_ describes the proportion of Notch-mediated control of the Hes1 promoter (Eqn. (S.4) of S1 Text). Fig 3 demonstrates the change in expression of *β*-catenin and *Hes1* as *θ*_2_ is varied from *θ*_2_ = 0.0 (regulation wholly Wnt-mediated), to *θ*_2_ = 1.0 (regulation entirely Notch-mediated). Simulations are presented for Wnt stimulus absent (e.g. crypt orifice, *W* = 0), present (e.g. crypt base, *W* = 1) or excess (e.g. hyperstimulated conditions, *W* = 2), aiming to mimic broad differences in the Wnt stimulus observed on ascending the intestinal crypt.

The simulations of this section explore how the relative strengths of Notch- and Wnt-mediated control of the Hes1 promoter in healthy cells affect Hes1 dynamics, and ultimately the cell fate outcome. This furthers our original aim of understanding how Notch-Wnt crosstalk influences the emergence of distinct cell fates.

#### Relative contribution of Notch- and Wnt-mediated action on the Hes1 promoter is key to shaping Hes1 dynamics

This study reveals how Hes1 expression changes as the system shifts from being in a Wnt-mediated to a Notch-mediated state. The relative strength of the two Hes1 regulation mechanisms strongly influences the dynamics of the Notch system. Oscillations occur where Hes1 transcription is substantially Notch-mediated (Fig 3D and 3E). When Wnt-mediated control dominates (Fig 3B) or the two routes are close to parity (Fig 3C), oscillations are more strongly damped and both cells settle rapidly on a constant steady state, characterised by reduced levels of Hes1.

Notably, the steady state of *β*-catenin appears unaffected by interaction with the Notch pathway, although some simulations at low-Wnt, high-*θ*_2_ develop small-amplitude oscillations about the steady state in response to the oscillations in the Notch system.

These results are consistent with the idea of Notch signalling maintaining flexibility in cell fate decisions [93]. Cells could regulate this by preserving a Notch-mediated monopoly of the Hes1 promoter, allowing Notch oscillations to persist until a *β*-catenin intervention at a specified time induces bistability and the commencement of fate selection.

#### Higher Hes1 levels correlate with reduced *β*-catenin

Inspection of individual cell pairs in Fig 3 identifies the expression patterns that emerge in the patterned, bistable state. Where slight differences emerge between the two cells’ *β*-catenin levels (Fig 3C–3E for *W* = 0, 1), higher Hes1 levels correlate with marginally lower *β*-catenin expression. This may be due to *β*-catenin being diverted for use in binding with NICD (Step ③ of Fig 2B) in the Notch-mediated route for Hes1 regulation. The stronger the Wnt stimulus, the more *β*-catenin is expressed, but the qualitative form of the *β*-catenin timecourse remains the same.

Cell fate selection in the intestinal crypt shows a high degree of spatial coordination, influenced in part by the variation in the strength of the extracellular Wnt stimulus. By looking at the variation in Hes1 and *β*-catenin expression with respect to *W*, we can begin to link the internal biochemistry of the cell with the external Wnt gradient characteristic to the crypt.

#### Notch-Wnt interaction supports spatial coordination in the crypt

By reading up the columns of Fig 3B–3E, we can see how the character of Hes1 and *β*-catenin expression changes as cells move up the crypt. We infer that oscillation of the Notch system is regulated by the extracellular Wnt stimulus. Hyperstimulation (*W* = 2) dampens oscillations and promotes homogeneity in Hes1 expression. This is due to the Wnt-dependent downregulation of Hes1 included in our model, representing Dsh-mediated activity at the Hes1 promoter. Furthermore, the transition from the Wnt-on (*W* = 1) state at the crypt base to the Wnt-off (*W* = 0) state at the orifice sees an increase in the maximum expression of Hes1 and an increase in the difference in Hes1 expression between each cell of the pair. These results are consistent with the emergence of cell heterogeneity in the intestinal epithelium as cells traffic up the crypt axis from a high to a low Wnt stimulus.

Notch-mediated control of the Hes1 promoter alone does not suffice to drive cell fate selection in the intestinal crypt. Rather, for Hes1 oscillations and heterogeneous cell fates to occur, we require coordination between Notch-mediated control of the promoter and a drop in the Wnt stimulus. Key to this coordination is the Dishevelled-mediated downregulation of Hes1 in the face of a high extracellular Wnt stimulus [48], which effectively creates a Wnt-dependent ‘sweet spot’ for Hes1 oscillations. This mechanism could offer an important role in linking the spatial gradients of extracellular Wnt to the coordination of emerging cellular heterogeneity within the intestinal crypt epithelium.

### Cell mutations are affected by Notch-Wnt crosstalk

All results presented thus far have focused on healthy cells. We now update the model to approximate the altered biochemistry of a ‘mutant’ or modified phenotype, with particular reference to the cells of the intestinal epithelium.

The first modified phenotype is an APC mutant, commonly implicated in colorectal cancer [108], in which the function of the *β*-catenin destruction complex is either partially or wholly impaired and therefore has a reduced binding affinity for *β*-catenin. APC is coded for genetically by two alleles, each of which is either healthy or mutated; the majority of CRC tumours display inactivation of both alleles [109]. APC is represented implicitly in our model, via the formation of the destruction complex *C*. We emulate APC mutation via a multiplier *ρ*_*APC*_ ∈ [0, 1], which scales the rate of formation of the destruction complex in Eqns. (S.9) and (S.10) for *G* and *C* respectively, detailed in S1 Text. Healthy cells possess two normal copies of the APC allele and therefore have *ρ*_*APC*_ = 1.0. A single-hit APC mutation, in which one allele is mutated, takes *ρ*_*APC*_ = 0.5, whilst a two-hit mutation sets *ρ*_*APC*_ = 0.0.

The second modified phenotype confers a hyperstimulated Wnt state and is motivated by suggestions in the literature that differential response to Wnt stimuli, rather than differential exposure, is a major influence upon cell proliferation [6]. The hyperstimulated phenotype is implemented by changing the value of the Wnt stimulus *W* for the affected cell from the reference state *W* = 1.0, to *W* = 2.0. This study aims to explore how variability in the response to local Wnt stimulus across a cell population might impact upon the expression of cell fate determinants such as Hes1.

**Fig 4:**
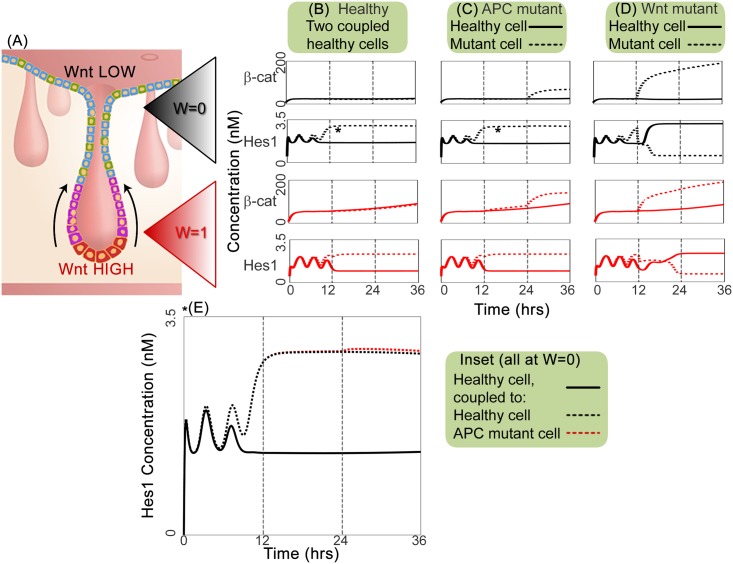
Dynamic timecourses showing cell pair responses to mutation in one cell. (A) Cross-section schematic of a crypt from the large intestine; image adapted from Reizel *et al*. [8], originally published by PLoS and provided under a Creative Commons Attribution Licence, *CC-BY-2.5*. (B–E) Timecourses for cell pairs started from homogeneous initial conditions; all cells are healthy at start of simulation. (B) Healthy cell pair; (C) APC mutants (dashed line) acquire their first APC knockout (*ρ*_*APC*_ = 0.5) at *t* = 12*h* and the second (*ρ*_*APC*_ = 0.0) at *t* = 24*h*; (D) hyperstimulated Wnt mutants (dashed line) transform to a *W* = 2.0 state at *t* = 12*h*. Except for Wnt mutants, all plots in panels (B)–(D) indicate simulations with (red) *W* = 1.0 and (black) *W* = 0.0. Inset panel (E) compares the Hes1 expression in the healthy and APC mutant scenarios for *W* = 0, indicated by the asterisks. In this case, the healthy scenario is shown in black and the APC mutant in red. Standard initial conditions and parameters are used, stated in Tables S.3–S.7 of S1 Text; the ODE model comprises equations (S.1)–(S.13) of S1 Text, with APC modifications to Eqns. (S.9) and (S.10) of S1 Text for study (C).

Results for two-cell simulations of these modified phenotypes are shown in Fig 4B–4E. In all cases except the hyperstimulated Wnt mutant, we fix *W* = 0.0 (Wnt stimulus off, black plots) or *W* = 1.0 (Wnt stimulus on, red plots), to simulate conditions near the crypt orifice or base respectively. Cell pairs are healthy at *t* = 0*h* and are initialised as for the previous two-cell simulations. In the APC mutant study, one cell acquires a single APC mutation at *t* = 12*h* and a second hit at *t* = 24*h*. For the Wnt mutant study, one cell mutates to a hyperstimulated state at *t* = 12*h*.

We begin by examining the first mutant type, in which APC is partially or wholly inactivated. These simulations, shown in Fig 4B, demonstrate the biochemical consequences of mutation and the impact upon cell fate.

#### Role of Notch-Wnt crosstalk in counteracting effects of APC mutation

Although the effects of APC mutation are most quickly apparent in the Wnt pathway, Notch-Wnt crosstalk can transmit this disturbance to the Notch pathway too. Crosstalk can also act as a cellular safeguard, which we demonstrate by simulating outcomes at three different external Wnt stimuli.

Fig 4C reveals that the first APC hit breaks the symmetry of the cells’ *β*-catenin dynamics and these differences are magnified once the second hit appears. The acquisition of APC mutation(s) reduces the rate of destruction of *β*-catenin, thereby increasing its expression within the cell; this effect is more pronounced for the high-Wnt conditions associated with lower regions of the crypt. Two mutations are required for a substantial departure from the healthy state.

Some small changes in Hes1 expression occur, as shown in the inset of Fig 4E, although these only become apparent after the second APC hit. Impairment of the destruction complex enables the mutant phenotype to express higher levels of Hes1, associated with prolonged mitotic activity, and reduced levels of Hath1, which promotes cell-cycle exit and is associated with further cell fate specification. Elevated levels of *β*-catenin in the APC mutant upregulate Hes1, via Notch- and Wnt-mediated routes. That this is more pronounced for *W* = 0 may indicate a role for the Dsh-mediated downregulation of Hes1 in counteracting the effects of mutation near the crypt base, when the local Wnt stimulus is high.

We now examine the second mutant type, which is hyper-responsive to the external Wnt stimulus.

#### Wnt hyperstimulation induces cell fate switching

These simulations indicate how crosstalk between Notch and Wnt can induce considerable changes in the Notch system as a result of a mutation which occurs in the Wnt pathway. Of particular interest are the Hes1 timecourses in Fig 4D, which show the Wnt mutation to a single cell causing opposite fates to be attained by both the healthy and mutant cell of the pair.

The initial timecourse for Hes1 in Fig 4D oscillates and demonstrates mild divergence by *t* = 12*h*, in both the upper (*W* = 0) and lower (*W* = 1) crypt. At this point, the second cell has the higher Hes1 expression. Following hyperstimulation of the second cell, there is an abrupt change in the pattern of Hes1 expression; Hes1 levels in the second cell fall sharply, while those in the healthy cell increase. Hes1 oscillations cease and the cells evolve to a constant steady state, with a high-Hes1 healthy cell and a low-Hes1 mutant. Hyperstimulation of the Wnt pathway enables the mutant cell to invert the cell fate decision of its neighbour, forcing it from a primary to a secondary fate. Although this behaviour is evident in both the Wnt-on and Wnt-off scenarios, the transitions post-mutation are more pronounced and occur over shorter times when *W* = 0. This reversal of roles affects all variables in the Notch submodel. The Wnt hyperstimulated mutants display substantially elevated *β*-catenin expression compared to the healthy case (Fig 4B): by *t* = 24*h*, a 2.2-fold increase at *W* = 1, and an 8.3-fold increase at *W* = 0.

The observed reversal of Hes1 expression patterns in the cell pair may have consequences for fate selection in a multicellular environment. Given that Hes1 expression is associated with maintaining a proliferative phenotype, the ability of a mutant cell to invert cell fate decisions could stimulate surrounding cells to continue in a mitotically active state for longer. The elevated *β*-catenin expression of hyperstimulated mutants would also help to maintain active cycling. Consequently, mutation events might generate mitotically active clusters which are only partly composed of aberrant cells, as for example in the Cancer Stem Cell Hypothesis [110, 111].

The healthy and mutant simulations of our model have highlighted how Notch-Wnt crosstalk shapes the internal cellular response to changes in the local environment. In particular, the Hes1 crosstalk hub has emerged as a key effector in resolving the relative input from the Notch and Wnt pathways and coordinating an appropriate cell fate response. This role may extend to resisting the effects of harmful mutations, as in the case of the APC mutant. In the discussion which follows, we unite these findings with our earlier observations from CRNT.

## Discussion

Our mathematical model for Notch-Wnt crosstalk captures the main qualitative features of each pathway, such as the Notch pathway’s capacity for damped oscillations and the Wnt pathway’s regulation of *β*-catenin expression by the extracellular Wnt concentration, and provides good agreement with the available experimental data [52, 104]. Computational exploration of our model, whether through the powerful abstractions of CRNT or the *in silico* simulation of cell pairs using a parameter- and dynamic-specific instantiation, has demonstrated how a nuanced balance of Notch- and Wnt-mediated regulation of the Hes1 promoter shapes the timing and outcome of cell fate selection in the intestinal crypt epithelium. The following principal findings have emerged through analysis and simulation of either homogeneous cell populations or heterogeneous cell pairs:

- **Wnt stabilises Notch:** Direct action of *β*-catenin on the Hes1 promoter confers a single steady state on a homogeneous Notch-Wnt network, dampening oscillatory dynamics in the Notch pathway. *β*-catenin crosstalk stabilises the output of the Notch pathway and reduces the flexibility of fate decision which would otherwise be conferred by oscillations in Hes1.
- **Relative contribution of Notch- and Wnt-mediated control of the Hes1 promoter shapes Notch dynamics:** The presence or absence of oscillations is associated with control of the Hes1 promoter. Notch-mediated regulation of Hes1 transcription promotes oscillations, while Wnt-mediated regulation via direct binding of *β*-catenin to the Hes1 promoter dampens oscillations and induces the cell to settle on a constant steady state. Furthermore, Wnt-induced downregulation of Hes1 via the interaction of Dishevelled with the Hes1 promoter may serve to prevent oscillations in regions where the Wnt stimulus is too high.
- **Role for Notch-Wnt crosstalk in counteracting the effects of APC mutation:** APC mutation impairs the action of the *β*-catenin destruction complex, increasing the expression of *β*-catenin and hence Hes1, via Notch- and Wnt-mediated transcription routes. Effects on Hes1 are marginally more pronounced in the low-Wnt conditions of the upper crypt. This may indicate a role for the Wnt-related downregulation of Hes1 via Dishevelled, in buffering the effects of mutations downstream of Dsh in the Wnt pathway, in the high-Wnt conditions of the lower crypt.
- **Wnt hyperstimulation can determine the fate of neighbouring cells:** Our simulations of cell pairs suggest that a Wnt-hyperstimulated cell may drive neighbouring cells to adopt a secondary, low-Delta fate.

We now discuss the biological implications of our findings. For example, cells might regulate the relative contribution of Notch- and Wnt-mediated transcription routes in order to coordinate the timing of cell fate selection. As cells migrate up the crypt, Wnt levels and hence *β*-catenin expression fall, enabling a shift towards Notch-mediated control of the Hes1 promoter and favouring the emergence of distinct cell fates.

Other features emerging from our model include the fate reversal seen in healthy cells neighbouring a Wnt-hyperstimulated phenotype, which could have some relevance to the cancer stem cell hypothesis, such that a hyperstimulated cell could maintain neighbouring healthy cells in a mitotically active state.

The success of chemical reaction network theory in identifying the emergence of full-system multistability from two monostable subnetworks highlights the importance of including pathway crosstalk in our mathematical models, if the richness of the underlying biochemistry is to be captured. Very different dynamics are obtained when the crosstalk between the Notch and Wnt pathways is accounted for, and our model has demonstrated that the Wnt pathway in particular has substantial capacity to influence the outcomes of Notch signalling. Crosstalk between the pathways should therefore be included in any future mathematical models where both proliferation and cell fate specification are being investigated.

**Fig 5:**
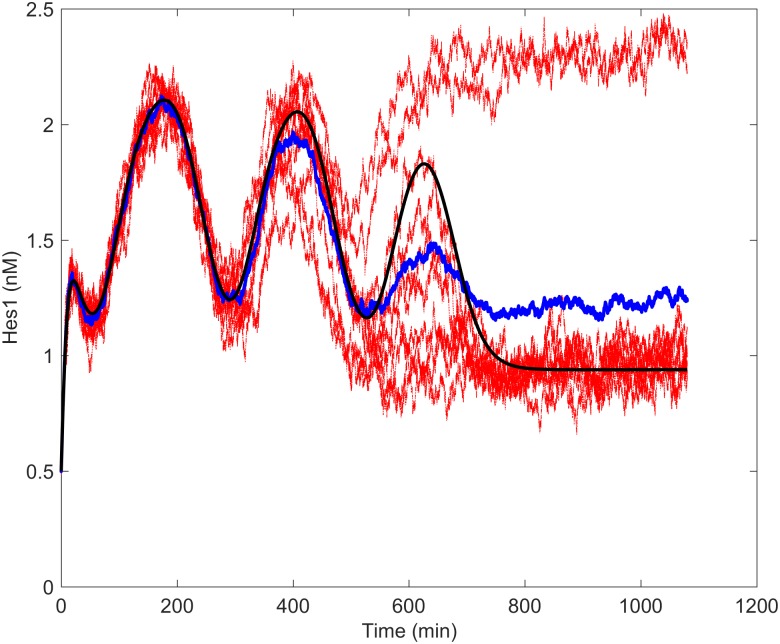
Proof-of-concept simulations for stochastic evolution of Hes1. The black timecourse indicates the outcome from the deterministic model; red lines indicate the results of ten separate stochastic runs, with mean given by the blue line. The addition of extrinsic noise to the Hes1 production term changes the outcome from a lower to a higher expression level of Hes1 in three out of the ten cases.

An inherent stochasticity in the production of Hes1 has been reported in the literature [66–68]. An interesting avenue of future work would be to include this stochasticity in our model. To illustrate this, we have produced a proof of concept simulation where we have added extrinsic noise to the Hes1 production term in Eqn. (S.4) of S1 Text. This is undertaken in the same manner as [112], where we have solved the equations with a fixed timestep and added normally distributed (*N*(0, *σ*^2^)) noise to Eqn. (S.4) of S1 Text at each timestep to represent a Wiener process. Fig 5 shows the results of 10 runs of such a simulation, with variance *σ* = 1. The addition of stochasticity allows individual solutions to go to either absorbing steady state; this is consistent with assertions that biochemical noise can influence cell fate [113]. Three of the ten stochastic simulations shown settle on the opposite state to the deterministic model; this is analogous to the cell adopting an absorptive, rather than a secretory, fate. A fuller exploration of stochastic effects would be a valuable area for further development of the model in future.

Simulations of cell pairs have yielded useful insights into healthy and aberrant scenarios. Future work would need to extend these studies to larger cell populations, preferably within a geometrically realistic crypt setting, to explore how the mutations described manifest at tissue level and over longer timescales. Full-crypt simulations (capturing three-dimensional populations of crypt cells, as for example in [114, 115]) to extend the Wnt hyperstimulation study might also examine whether the cancer stem cell hypothesis emerges from our model in larger populations. Given our focus upon the Hes1 promoter, it may prove beneficial to refine this area of the model to incorporate Hes1 mRNA and dimerisation as in other mathematical models [79, 80, 85, 86], to enable closer matching of the oscillatory readings with experimental data or, alternatively, refinement of the Wnt submodel to include explicit representation of Dishevelled [24, 32]. Furthermore, the analysis and simulation of the deterministic model discussed here provide a foundation from which to explore the effects of stochastic expression of Hes1; this could prove a valuable extension of the model and improve the matching of Hes1 expression to experimental data.

## Methods and models

### ODE solvers

Numerical solution of ODEs, involved in the steady-state analysis and all cell pair simulations, is perfomed in Matlab, using the software’s own suite of solvers [116]. Owing to the stiffness of the ODE model, we employ the solver ode15s: this is a multistep, variable order solver, and employs an algorithm based upon the numerical differentiation formulas [116].

### Chemical reaction network theory analysis

All CRNT analyses are performed using the *Chemical Reaction Network Toolbox*, a computational package for analysing the stability properties of chemical reaction networks, indicating whether a given network is capable of multiple stable states, or only one [100]; it is a stand-alone package, purpose-built for CRNT analysis. The toolbox requires the user to specify the details of each reaction in the network of interest. Each inclusion takes the form $\text{A}+\text{B}\overset{\text{E}}{\to }2\text{C}+\text{D}$, where A and B are the reactants, C and D are the products, and E is either an inhibitor or a promoter of the reaction.

We supply the toolbox with the species and network connectivity of the Notch subnetwork, Wnt subnetwork and fully coupled network (Fig 2), along with the *influence specification*, the species which up- or down-regulate each interaction. We also provide two versions of the Notch subnetwork and whole network. The first version contains both Wnt- and Notch-mediated regulation of Hes1 (Steps ⑭ and ④ of Fig 2 respectively), while the second involves only Notch-mediated control. The toolbox also requires stoichiometry information; for instance, specifying 2C rather than just C as a product in the above example. However, the kinetics of each reaction (i.e. the functional forms of the reaction rates) and parameter values do not need to be specified: the toolbox would not require a specific functional form for the dependence of the reaction rate upon E in the above example. The toolbox performs a sign-checking operation on two quantities derived from the network’s stoichiometry [100] and classifies the network according to its ability to exhibit multistationarity if allied with particular types of kinetics.

It is not possible to analyse states of the heterogeneous system, owing to the excessively large computation time for a system of this complexity; consequently, all our CRNT results relate to a homogeneous Notch pathway (in which we assume $D=\bar{D}$ in Eqn. (S.1) of S1 Text). Crosstalk species in the decoupled systems are represented as full reactants with their own inflows and outflows.

### Parametrisation

Computational implementation of our model requires us to determine appropriate parameter values. Some of our 41 parameters, such as decay rates, are readily amenable to experimental measurement, whilst others, such as binding rates, are not. Experimental estimates are not available for 21 of our model parameters at the present time. Consequently:

1. Estimates derived from human cell lines, in particular intestinal epithelial lines, have been used wherever possible (5 cases);
2. Where data from human cell lines is absent, values from mammalian lines have been employed where possible (5 cases);
3. Otherwise, non-mammalian readings (4 cases) or values from published mathematical models (21 cases) have been used as initial estimates for parameter fitting studies.

The remaining six parameters are exponents for the Notch model and are derived from observations of a similarly-structured model [117] and an understanding of the qualitative behaviour displayed by the biological regime.

Our primary focus is on the qualitative features of the model within biologically realistic regimes. Nonetheless, it is hoped that qualitative predictions from our model could stimulate future experimental estimation of its parameters within a single, human intestinal cell line. Parameter estimates resulting from computational fitting are assumed to represent a population average; the non-compartmental nature of our model assumes that reactant species are present at a uniform concentration throughout the cell.

All parameter fitting uses the decoupled Notch and Wnt systems. In each case, an initial set of parameter values is formed, applying criteria 1–3 above. A sensitivity analysis is performed on this set using the Systems Biology Toolbox, an add-on kit for Matlab [116, 118], to determine the order in which parameters are to be sequentially varied. The sensitivity, *S*_*k*_ of *X*, where *X*(*k*) is either the *β*-catenin steady state B* or the Hes1 oscillation period *T*, to a given parameter *k* is defined by the following formula:

$$S_{k}\;=\;\frac{\left|X\left(k\;+\;\delta_{k}\right)\;-\;X\left(k\right)\right|}{\delta_{k}},$$

where *δ*_*k*_ is the incremental change in the parameter *k*, for the given parameter set using parameter *k*. This is converted into the *normalised sensitivity index*, *NS*_*k*_, by

$$NS_{k}\;=\;\frac{k}{X(k)}\;\times \;S_{k}.$$

Normalised sensitivities are used to create a parameter priority ordering, with the most sensitive parameters fitted first.

Analysis of the decoupled system reveals the most sensitive parameter of the *β*-catenin steady state B* is *α*_4_ (a parameter involved in rate of production of *β*-catenin subject to Wnt stimulus *W*) whereas the parameter to which period of Hes1 oscillations is most sensitive is *κ*_7_ (a dissociation rate constant involved in Wnt-mediated transcription of Hes1). A summary of the sensitivity results is presented in Fig. S.4 of S1 Text.

**Fig 6:**
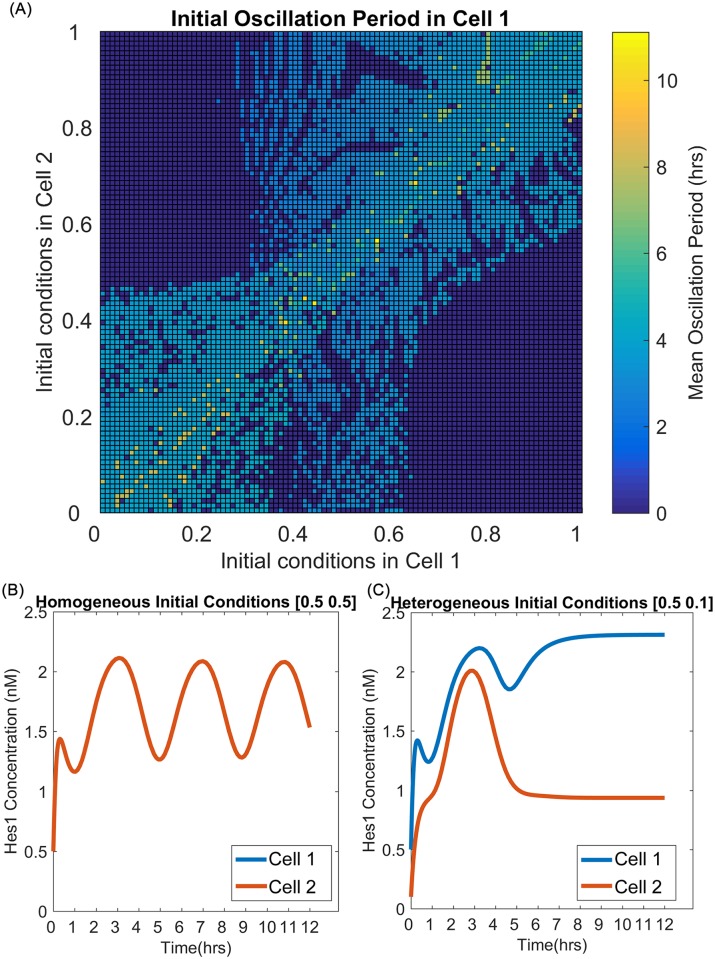
Notch parametrisation outcomes. (A) Response of oscillation period in cell 1 to variations in initial conditions in a two-cell system running the decoupled, dimensional Notch model, Eqns.(S.1)–(S.6) of S1 Text. Statements of initial conditions of the form [*x*, *y*] indicate that all seven Notch species in cells 1 and 2 are initialised to *x* and *y* respectively. Owing to symmetry considerations, only the oscillations in Cell 1 were measured; results for Cell 2 correspond to a reflection of this surface in the line *y* = *x*. An amplitude filter was applied during generation of the plot, to disregard any small-amplitude oscillations (< 0.001) arising from the computational solution process, rather than true oscillations of the ODE model. (B) Diagonal entries of (A) yield homogeneous evolution with damped oscillations, as in this timecourse of a cell pair from initial conditions (0.5, 0.5). (C) Off-diagonal entries of (A) show heterogeneous evolution, as in this timecourse of a cell pair from initial conditions (0.1, 0.5).

**Fig 7:**
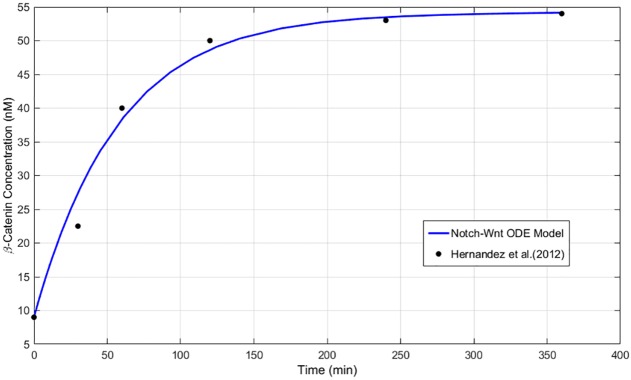
Wnt parametrisation outcome. Timecourse for *β*-catenin evolution in the dimensional, decoupled Wnt submodel, Eqns. (S.9)–(S.13) of S1 Text (line graph), compared against the experimental readings of Hernández *et al*. [104] (point data). The timecourse for the Wnt submodel uses the parameter values listed in Tables S.3–S.6 of S1 Text, and the initial conditions of Table S.7 of S1 Text.

Parameter fitting is performed using Matlab; a one-dimensional approach is used, in which each parameter in turn is modified over 1000 evenly spaced values within a ±100% tolerance while the other parameters are held constant, and aiming to remain at a biologically plausible order of magnitude. As a given parameter is varied, the resulting model output is measured against the target data (Hes1 oscillation period in the Notch system, *β*-catenin steady state in the Wnt system) and the new value selected which delivers the closest match. Parameters are modified sequentially on a loop until the model output lies within a given tolerance of the target data. Although this approach to obtaining a parameter set is unlikely to yield the global optimum, it nonetheless provides a parametrisation which is fully grounded in the literature, biologically plausible, and results in biologically plausible behaviour, as evidenced by the matching to experimental data shown in Figs 6 and 7.

Parametrisation of the Notch submodel defined by Eqns. (S.1)–(S.7) of S1 Text targets the two hour period of Hes1 oscillations observed by Hirata *et al*. [52] in murine myoblast cells, averaged over a twelve hour period following initial stimulation. Initial parameter estimates were determined by solving for the homogeneous state of a two-cell system. Data from the model of Shepherd [117] were used to locate an oscillatory regime of our Notch model, yielding suitable initial estimates for missing parameters, while initial conditions for model variables were chosen to be of the same order as in the model of Agrawal *et al*. [85]. Exponents for Hill functions and hyperbolas are set to *m*, *n* = 3. This reflects the strength of feedback required to generate oscillations in the absence of a delay-driven formulation; exponents are not included in the parameter fitting exercise. On completion of the fitting procedure, the parameter set was tested in a two-cell, heterogeneous system and the oscillation period measured over a range of starting conditions. Where oscillations occur, the period generally lies within the 2–4 hour range (Fig 6). This offers a reasonable match to the data of Hirata *et al*. but tends to overestimate the oscillation period, as expected for a non-delay model of this kind [77, 79]. Such modifications as dimerisation or stochasticity might provide the basis for more extensive investigation in the future but lie outside the scope of the present paper.

Parametrisation of the Wnt submodel described by Eqns. (S.9)–(S.13) of S1 Text uses the data of Hernández *et al*. [104], which supplies a *β*-catenin timecourse for the human colon carcinoma cell line, *RKO*. Consequently, all Wnt stimuli in our model are nondimensionalised against a reference value of 100 ng/ml. The unstimulated state, *W* = 0 in our model, equates to 0 ng/ml; the reference value represents *W* = 1. All other values scale linearly with this, with values *W* > 1 representing a hyperstimulated state. Steady-state data from Hernández *et al*. [104] are used to generate a pair of simultaneous equations (Eqns. (S.33), (S.34)) which supply estimates for two unknown parameters, *α*_3_, *α*_4_. Thereafter we fit the three-hour time course for *β*-catenin. The mean squared error (MSE) of the model’s performance, $\hat{X}$, against the experimental data, *X*, is calculated in each case:

$$MSE\;=\;\frac{1}{n}\sum_{1}^{n}{\left(\hat{X}\;-\;X\right)}^{2}\;,$$

where *n* = 6, the total number of observations. Estimates for the initial concentrations of reactant variables are drawn either from the experimental work of Tan *et al*. [35] which uses three human cell lines, or from the mathematical model of Lee *et al*. [24], based on *Xenopus* oocytes. The resulting *β*-catenin evolution of our Wnt submodel, shown in Fig 7, provides a close fit to the data of Hernández *et al*. [104].

In general our parameter values are of the same order of magnitude as those of other Notch and Wnt models in the literature [24, 27, 77, 85]. Key differences occur where our estimates benefit from more recent experimental data [104], for example in adopting the *β*-catenin decay rate *μ*_*B*_ = 6.36 × 10^−6^ min^−1^ rather than the *μ*_*B*_ = 2.57 × 10^−4^ min^−1^ used elsewhere [28].

Comparisons of our decoupled models against experimental data (see Figs 6 and 7) indicate that they capture the qualitative features of the Notch and Wnt systems and do so within a biologically sound regime. This enables us to apply the full, coupled model to problems of biological and biochemical interest. Experimental refinement of some of the parameters might also facilitate a global analysis of the parameter space, and render the model suitable for quantitative predictions in the future.

## Supporting information

S1 Text Model Development and Implementation. This supplementary file presents a more detailed model development for our Notch-Wnt ODE model. This includes: full listing of the model equations; details of steady-state analyses for the Notch and Wnt submodels; additional information about parametrisation; and tables detailing the values and origins of the model parameters. This text also provides a technical outline of chemical reaction network theory.
(PDF)
