# Supplementary material for: The role of the Hes1 crosstalk hub in Notch-Wnt interactions of the intestinal crypt
Source: PLoS Comput Biol. 2017 Feb 28;13(2):e1005400. doi: 10.1371/journal.pcbi.1005400 (PMC5363986; doi:10.1371/journal.pcbi.1005400)
Supplement: S1 Text — This supplementary file presents a more detailed model development for our Notch-Wnt ODE model. This includes: full listing of the model equations; details of steady-state analyses for the Notch and Wnt submodels; additional information about parametrisation; and tables detailing the values and origins of the model parameters. This text also provides a technical outline of chemical reaction network theory. (PDF) [file pcbi.1005400.s001.pdf]

Supplementary Information

The following supplementary information details our mathematical model for Notch-Wnt interaction, through a statement of the twelve differential equations alongside the associated experimental evidence. All numbered steps coincide with the numbering of Fig. 2 of the main text. We also introduce the parameters associated with the dimensional version of our model, and outline the steady state analysis of the decoupled systems. The ODEs (S.1) – (S.13) comprise our model of Notch-Wnt interaction for use in the cell pair simulations.

Model Development

We now describe the ODEs which comprise our Notch-Wnt interaction network. With a slight abuse of notation, we denote by X "the concentration of reactant X", rather than $[X]$.

The system comprises twelve ODEs: six for the Notch pathway, four for the Wnt pathway, and two for intermediate complexes which either mediate interactions between the two pathways (I_1) or which respond to the strength of extracellular Wnt signalling to regulate the level of active β-catenin in the cell (I_2). The dependent variables, abbreviating letters and associated parameters can be found in Tables S.1 and S.2 respectively.

We represent the extracellular Wnt stimulus by a time-dependent, nondimensional quantity, $W(t)$; the simulations presented here use only scalar values $W = 0, 1, 2$, but there is scope for exploring time-varying Wnt stimuli in future. Other nondimensional variables and parameters are indicated by the dash notation $'$, except where specified otherwise.

Notch Pathway Submodel

Our submodel for the Notch pathway is shown in Fig. 2B of the main text, and includes both receptor-ligand binding at the cell surface membrane and subcellular details of Hes1 regulation. Its seven dependent variables are: Notch receptor, $N(t)$; Notch Intra-Cellular Domain (NICD), $F(t)$; intermediate 1, $I_1(t)$, representing NICD bound to β-catenin; Hes1, $H_1(t)$; proneural protein (Ngn3), $P(t)$; Delta ligand, $D(t)$; and Hath1 $H_2(t)$, as detailed in Table S.1.

The use of Hill-type and hyperbolic functional forms in the Notch submodel follows a four-component, non-delay model by Shepherd [1], itself an adaptation of a delay model due to Momiji and Monk [2]. Shepherd's model is capable of generating oscillations from a non-delay formulation.

Notch receptor, N. We assume that the dominant processes regulating levels of Notch are its production and fragmentation. Production of Notch receptor is modelled by a Hill function, similar in form to the rates suggested by Shepherd [1] and Collier *et al.* [3]. The linear decay term represents natural decay of Notch, as well as its fragmentation to release NICD:

$$\frac{dN}{dt} = -\mu_N N + \underbrace{\frac{\theta_1 \bar{D}^{m_1}}{\kappa_1^{m_1} + \bar{D}^{m_1}}}_{\text{Steps (1) and (9)}}, \tag{S.1}$$

where $\bar{D}$ is the mean Delta level expressed by neighbouring cells and μ_N the rate of ligand fragmentation, assumed constant. The Hill function has dissociation constant κ_1,

Variable	Description	Units	Scaling
t	Time	min	$\tau = \mu_N t$
N	Membrane-bound Notch receptor	nM	$N = \frac{\theta_1}{\mu_N} N'$
F	Notch Intracellular Domain (NICD)	nM	$F = \frac{\theta_1}{\mu_F} F'$
H_1	Hes1	nM	$H_1 = \frac{\xi_2}{\mu_{H_1}} H_1'$
P	Ngn3	nM	$P = \frac{\xi_3}{\mu_P} P'$
D	Delta ligand	nM	$D = \frac{\theta_4}{\mu_D} D'$
H_2	Hath1	nM	$H_2 = \frac{\xi_5}{\mu_{H_2}} H_2'$
G	GSK3β	nM	$G = \frac{\theta_1}{\mu_G} G'$
C	Destruction complex	nM	$C = \frac{\theta_1}{\mu_C} C'$
B	Active β-catenin	nM	$B = \frac{\theta_1}{\mu_N} B'$
A	Axin	nM	$A = \frac{\theta_6}{\mu_A} A'$
I_1	Intermediate 1 (NICD/β-catenin)	nM	$I_1 = \frac{\theta_1}{\mu_{I_1}} I_1'$
I_2	Intermediate 2 (GSK3β/β-catenin)	nM	$I_2 = \frac{\theta_1}{\mu_{I_2}} I_2'$

Table S.1. Variable listings for our coupled Notch-Wnt ODE model. The independent variable, time, is presented first, followed by the twelve dependent variables representing network reactants.

maximal rate θ_1 and Hill coefficient m_1.

Notch Intracellular Domain (NICD), F. For simplicity, we treat the three cleavages of the Notch receptor as a single event. NICD is a fragment of the membrane-bound Notch receptor and so its production is assumed to scale with the rate of Notch fragmentation, as $\alpha_{\text{frag}}\mu_N N$. The nondimensional constant α_{frag} represents the proportion of Notch which results in generation of NICD; it is determined via parameter fitting against experimental data, as described in Methods and Models. $\alpha_{\text{frag}} < 1$ acknowledges that loss of Notch in Eqn. (S.1) is due to natural decay as well as NICD fragmentation. The rate of removal of NICD is assumed to depend on the rate at which it binds with β-catenin, with rate constant α_1, along with natural decay at rate μ_F. These assumptions for production and loss of NICD yield the following ODE:

$$\frac{dF}{dt} = -\mu_F F + \underbrace{\alpha_{\text{frag}}\mu_N N}_{\text{Step } ②} - \underbrace{\alpha_1 B \cdot F}_{\text{Step } ③} \,. \tag{S.2}$$

Intermediate 1, I_1. Our model assumes the evolution of I_1 to be governed by its formation from β-catenin and NICD, and its dissociation. In the absence of suitable experimental data, we assume a 1-1 stoichiometry for the binding of β-catenin and NICD to form I_1 ($B + F \rightleftharpoons BF\,(= I_1)$). Given the strong experimental evidence for such binding, we assume that the rate at which the reverse reaction occurs is negligible. A first-order mass action law is then used to derive the reaction rate for intermediate formation, namely $\alpha_1 B \cdot F$. Dissociation of I_1 adopts a simple linear form, with decay

rate constant μ_{I_1}. Combining these considerations yields the following ODE for I_1:

$$\frac{dI_1}{dt} = -\mu_{I_1} I_1 + \underbrace{\alpha_1 B \cdot F}_{\text{Step } ③} . \tag{S.3}$$

Hes1, H_1**.** In our model, Hes1 is subject to transcriptional regulation by B, I_1, H_1 and (implicitly) *Dsh* and its transcription is assumed to have a maximal rate, ξ_2. B and I_1 are assumed to be independent *upregulators*, modelled via Hill functions with exponents m_2, m_7 and Hill coefficients κ_2, κ_7 (see Eqn. (S.4)), with an additive effect upon the promoter, owing to the separate promoter binding sites known to exist for I_1 and β-catenin [4]. The relative contributions of I_1 and B to the upregulation are described by the non-negative, nondimensional constants θ_2, θ_7, such that $\theta_2 + \theta_7 = 1.0$. This constrains the sum of the two Hill functions to lie in the range $[0, 1]$ and reflects our assumption that all upregulation of Hes1 is either Notch-mediated or Wnt-mediated.

Downregulators of Hes1 transcription are H_1 (i.e. autorepression) and *Dsh*. The autorepression is modelled by a hyperbola in H_1, with exponent n_2 and inhibition constant σ_2. For simplicity we do not represent Dsh explicitly in our model and assume instead that it is a decreasing function of Wnt, which we denote Ψ_W:

$$\Psi_W = \Psi(W(t)) \equiv \frac{\sigma_K}{\sigma_K + W(t)} ,$$

where σ_K is an inhibition constant to be specified. Ψ_W attenuates the expression of Hes1 in response to a strong extracellular Wnt stimulus.

Combining these assumptions and assuming linear decay of Hes1 yields the following ODE for its evolution:

$$\frac{dH_1}{dt} = -\mu_{H_1} H_1 + \Psi_W \left(\underbrace{\frac{\theta_2 I_1^{m_2}}{\kappa_2^{m_2} + I_1^{m_2}}}_{\text{Step } ④} + \underbrace{\frac{\theta_7 B^{m_7}}{\kappa_7^{m_7} + B^{m_7}}}_{\text{Step } ⑭} \right) \underbrace{\frac{\xi_2 \sigma_2^{n_2}}{\sigma_2^{n_2} + H_1^{n_2}}}_{\text{Step } ⑤} , \tag{S.4}$$

where μ_{H_1} is the rate of Hes1 decay.

Proneural protein, P**.** We account for the transcriptional inhibition of Ngn3 by Hes1, via a hyperbola in H_1, with maximal rate ξ_3, exponent n_3 and inhibition constant σ_3. If we assume further that Ngn3 undergoes natural decay, we obtain the following ODE for its evolution:

$$\frac{dP}{dt} = -\mu_P P + \underbrace{\frac{\xi_3 \sigma_3^{n_3}}{\sigma_3^{n_3} + H_1^{n_3}}}_{\text{Step } ⑦} , \tag{S.5}$$

where μ_P is the rate of decay of proneural protein.

Delta ligand, D**.** For simplicity, we consider only Delta (rather than Jagged) as the binding partner in our model; nor do we distinguish between the three distinct types of Delta ligand known to exist in mammals [5].

Following Shepherd [1], we model the synthesis of Delta by a Hill function in P, with maximal rate θ_4, Hill coefficient m_4 and dissociation constant κ_4. If we assume further that Delta undergoes natural decay at rate μ_D then we obtain the following ODE for its evolution:

$$\frac{dD}{dt} = -\mu_D D + \underbrace{\frac{\theta_4 P^{m_4}}{\kappa_4^{m_4} + P^{m_4}}}_{\text{Step } ⑧} . \tag{S.6}$$

We note that, in a given cell, the reaction cascade running from Notch to Delta via Hes1 serves to downregulate Delta when that cell is expressing high levels of Notch. For this reason we expect either Step ① or Step ⑨ to dominate in any one cell, in cases where the cascade is driving lateral inhibition or cell type segregation.

Hath1, H_2**.** We use a hyperbola to model the inhibitory influence of Hes1 upon the production of Hath1, with maximal rate ξ_5, exponent n_5 and inhibition constant σ_5. If we assume further that Hath1 decays linearly with rate constant μ_{H_2}, then we obtain the following ODE for H_2:

$$\frac{dH_2}{dt} = -\mu_{H_2} H_2 + \underbrace{\frac{\xi_5 \sigma_5^{n_5}}{\sigma_5^{n_5} + H_1^{n_5}}}_{\text{Step ⑥}} . \tag{S.7}$$

We note that H_2 decouples from the rest of the system; we retain Eqn. (S.7) nonetheless, as it provides a read-out for cell fate specification.

Eqns. (S.1) – (S.7) constitute our submodel for the Notch pathway. Crosstalk between the Notch and Wnt pathways centres upon NICD and Hes1, as we elucidate below when we describe our submodel for the Wnt pathway.

Wnt Pathway Submodel

In the interest of focusing upon the dynamics surrounding the Hes1 crosstalk hub, we present a pared-down representation of the Wnt system (Fig. 2A of the main text). Our model does not explicitly account for the evolution of phosphorylated β-catenin, which is degraded by the proteasome without participating in any other reactions.

Note

The literature offers several detailed models of the Wnt pathway, e.g. [6–8]. Our focus on Notch-Wnt crosstalk motivates the use of a less detailed Wnt model. This aims to capture the qualitative behaviour of major Wnt pathway species, such as GSK3β and β-catenin, as demonstrated by the fitting of the β-catenin concentration to data from Hernández *et al.* in Fig. 7 of the main text.

In developing the Wnt submodel, analytic forms for the steady states of the Wnt species were determined using Eqns. (S.9) – (S.13). The functional form of terms for β-catenin synthesis ($(1 + W(t))\alpha_4$), GSK3β synthesis ($(1 + W(t))\alpha_2$) and formation of the destruction complex ($\Psi_{W,A}$) were determined by inspection of the steady state expressions. The Wnt-dependence of these terms was chosen such that the response of the steady states to changes in Wnt stimulation showed qualitative agreement with the literature (e.g. β-catenin levels are enhanced by Wnt stimulation). Values for the exponents and multiplying parameter of $\Psi_{W,A}$ are determined by matching the Wnt response of the system to the experimental data of Hernández *et al.* [9], as described in *Methods and Models - Parametrisation.* Future work might refine the Wnt submodel to incorporate other species and relax the Wnt-dependence of the synthesis terms.

GSK3β**,** G**.** In our model, we consider a general "destruction complex" rather than accounting for all of its component parts, and model the transition of GSK3β between the complexed and non-complexed states as a reversible reaction, $G \rightleftharpoons C$. This may be an oversimplification, given assertions in the literature that the concentration of Axin within the complex may have a central role in regulating the rate of this step in

Xenopus oocytes [6]; however, *in vitro* experiments suggest that this is not the case for mammalian cell lines [10]. For simplicity, our model does not distinguish between the various spatially sequestered forms of GSK3β.

Owing to its dependence upon the local Wnt stimulus and the cellular Axin levels, we abbreviate the rate function for the forward reaction to $\Psi_{W,A}$. Applying the approach described in the above Note, we suppose the forward reaction $G \to C$ to be Wnt- and Axin-dependent, of rate

$$\Psi_{W,A} = \Psi(W(t), A(t)) \equiv \frac{1.4A(t)^2}{1 + (1 + W(t))^4}\,. \tag{S.8}$$

That is, the rate increases with Axin levels and decreases with Wnt stimulus. The reverse reaction $C \to G$ is assumed to occur at constant rate μ_C. The constant 1.4 is determined from the parameter fitting procedure described in the main text and in the above *Note*.

In our model, the loss of GSK3β arises from linear decay and transfer to the β-catenin destruction complex, C. We assume that there is a basal rate, α_2, of production of GSK3β, and that this rate increases when there is a Wnt stimulus, $W(t)$. This is an artefact of having a small-scale Wnt model, being required to yield suitable steady-state behaviour in response to Wnt stimulation; refinement of this aspect of the model is a possible area for future work.

We assume further that GSK3β binds reversibly with other proteins to form the destruction complex; these are assumed to be abundant. Combining these processes and assuming further that GSK3β undergoes natural decay, we deduce that its evolution can be written as:

$$\frac{dG}{dt} = -\,\mu_G G + (1 + W(t))\alpha_2 \underbrace{+\mu_C C - \alpha_5 \Psi_{W,A} G}_{\text{Step } (13)}, \tag{S.9}$$

where μ_G is the decay rate of G, μ_C the rate of dissociation of the destruction complex C and α_5 the rate constant for GSK3β incorporation into the destruction complex.

Destruction Complex, C. In our model, the destruction complex, C, is produced and lost at rates $\Psi_{W,A}$ and μ_C respectively, in the reverse manner to that of Eqn. (S.9). We assume further that the destruction complex is released from the intermediate I_2 at rate μ_{I_2}, and that it binds to β-catenin at rate α_3. Combining these processes, we deduce that the time evolution of C can be written as:

$$\frac{dC}{dt} = \underbrace{-\mu_C C + \alpha_5 \Psi_{W,A} G}_{\text{Step } (13)} + \mu_{I_2} I_2 - \underbrace{\alpha_3 B \cdot C}_{\text{Step } (11)}, \tag{S.10}$$

where $\Psi_{W,A}$ is defined as described above for GSK3β in Eqn. (S.9).

β-catenin, B. In our model, we assume that β-catenin is produced at a basal rate, α_4, which is enhanced when a Wnt stimulus is present. Loss of β-catenin arises from its binding to either NICD or the destruction complex, at rates α_1 and α_3 respectively, to form the intermediates I_1, I_2; natural decay of β-catenin is also assumed, at rate μ_B. Combining these assumptions, we obtain the following evolution equation for β-catenin:

$$\frac{dB}{dt} = -\,\mu_B B + (1 + W(t))\alpha_4 - \underbrace{\alpha_1 B \cdot F}_{\text{Step } (3)} - \underbrace{\alpha_3 B \cdot C}_{\text{Step } (14)}\,. \tag{S.11}$$

Intermediate 2, I_2. Intermediate 2 arises from the binding of the destruction complex to β-catenin. As for intermediate I_1, we assume that the forward reaction dominates and follows first-order mass action, whilst the dissociation of I_2 has rate constant μ_{I_2}. These assumptions yield the following ODE for I_2:

$$\frac{dI_2}{dt} = -\mu_{I_2} I_2 + \underbrace{\alpha_3 B \cdot C}_{\text{Step } (11)} . \tag{S.12}$$

Axin, A. We model Axin synthesis using a Hill function which depends upon B, with maximal rate θ_6, Hill coefficient m_6 and dissociation constant κ_6. Assuming further that Axin decays linearly at rate μ_A, we have:

$$\frac{dA}{dt} = -\mu_A A + \underbrace{\frac{\theta_6 B^{m_6}}{\kappa_6^{m_6} + B^{m_6}}}_{\text{Step } (10)} . \tag{S.13}$$

Axin presents in two functionally equivalent forms, Axin1 and Axin2 [11], but for simplicity we do not differentiate between these in our model.

Initial conditions for the dimensional model are specified in Table S.7.

Nondimensionalised System

Eqns. (S.1) - (S.13) define our ODE system in dimensional form; we shall now nondimensionalise it to reduce the number of system parameters and to facilitate estimation of the relative importance of the different reactions within the network.

Time is scaled against μ_N^{-1}, the timescale for decay of the Notch receptor; we define a nondimensional time, τ, such that $\tau = \mu_N t$. Scalings for dependent variables are introduced in Table S.1, and those for parameters in Table S.2. For reactant X, we also introduce the dimensionless parameter $\nu_X = \mu_X/\mu_N$ to represent the ratio of its decay rate to that of Notch. The twelve ODEs for the nondimensional system are as follows (primes denote dimensionless variables):

$$\frac{dN'}{d\tau} = -N' + \frac{\bar{D}'^{m_1}}{\kappa_1'^{m_1} + \bar{D}'^{m_1}}, \tag{S.14}$$

$$\frac{dF'}{d\tau} = \nu_F \left\{ -F' + \alpha_{\text{frag}} N' - \frac{\alpha_1'}{\nu_F} B' \cdot F' \right\}, \tag{S.15}$$

$$\frac{dI_1'}{d\tau} = \nu_{I_1} \left\{ -I_1' + \frac{\alpha_1'}{\nu_F} B' \cdot F' \right\}, \tag{S.16}$$

$$\frac{dH_1'}{d\tau} = \nu_{H_1} \left\{ -H_1' + \Psi_W \left(\frac{\theta_2 I_1'^{m_2}}{\kappa_2'^{m_2} + I_1'^{m_2}} + \frac{\theta_7 B'^{m_7}}{\kappa_7'^{m_7} + B'^{m_7}} \right) \frac{\sigma_2'^{n_2}}{\sigma_2'^{n_2} + H_1'^{n_2}} \right\}, \tag{S.17}$$

$$\frac{dP'}{d\tau} = \nu_P \left\{ -P' + \frac{\sigma_3'^{n_3}}{\sigma_3'^{n_3} + H_1'^{n_3}} \right\}, \tag{S.18}$$

$$\frac{dD'}{d\tau} = \nu_D \left\{ -D' + \frac{P'^{m_4}}{\kappa_4'^{m_4} + P'^{m_4}} \right\}, \tag{S.19}$$

$$\frac{dH_2'}{d\tau} = \nu_{H_2} \left\{ -H_2' + \frac{\sigma_5'^{n_5}}{\sigma_5'^{n_5} + H_1'^{n_5}} \right\}, \tag{S.20}$$

$$\frac{dG'}{d\tau} = \nu_G \left\{ -G' + C' + (1 + W(\tau))\alpha_2' - \alpha_5' \Psi_{W,A'} G' \right\}, \tag{S.21}$$

$$\frac{dC'}{d\tau} = \nu_C \left\{ -C' + I_2' + \alpha_5' \Psi_{W,A'} G' - \frac{\alpha_3'}{\nu_C} B' \cdot C' \right\}, \tag{S.22}$$

$$\frac{dB'}{d\tau} = \nu_B \left\{ -B' + (1 + W(\tau))\alpha_4' - \frac{\alpha_1'}{\nu_F} B' \cdot F' - \frac{\alpha_3'}{\nu_C} B' \cdot C' \right\}, \tag{S.23}$$

$$\frac{dI_2'}{d\tau} = \nu_{I_2} \left\{ -I_2' + \frac{\alpha_3'}{\nu_C} B' \cdot C' \right\}, \tag{S.24}$$

$$\frac{dA'}{d\tau} = \nu_A \left\{ -A' + \frac{B'^{m_6}}{\kappa_6'^{m_6} + B'^{m_6}} \right\}. \tag{S.25}$$

For brevity, we hereafter drop the prime notation from our nondimensional variables.

Model Parameters

Dimensional values for all parameters, along with supporting references, are shown in the following tables (listed in full at end of document):

- **Half-lives and decay rates**: Table S.3;
- **Hill parameters**: Table S.4;
- **Hyperbola parameters**: Table S.5;
- **Other parameters**: Table S.6;
- **Initial conditions**: Table S.7.

Suitable experimental data are not currently available to estimate values of all model parameters. Where appropriate data were lacking, parameter estimation was performed as described in Methods and Models (Parametrisation); numerically fitted values are indicated by a '*PF*' and/or footnotes.

Steady-state analysis

Analysis of Notch submodel

Decoupling Notch from the Wnt system leaves the system shown in Fig. 2B of the main text, represented by the ODEs (S.14) – (S.20). For clarity, we abbreviate the Hill functions and hyperbolas as follows:

$$\Phi_i(X) = \frac{X^{m_i}}{\kappa_i^{m_i} + X^{m_i}}, \quad \Theta_i(X) = \frac{\sigma_i^{n_i}}{\sigma_i^{n_i} + X^{n_i}}\,,$$

where all parameters are defined as in Table S.2 and $X \equiv X(\tau)$ is a reactant concentration at time τ. In what follows, we exploit the fact that Φ is monotonic increasing and Θ monotonic decreasing on $\mathbb{R}^+$, and hence that $\frac{d\Theta(X)}{dX} < 0 < \frac{d\Phi(X)}{dX}$, $\forall X \in \mathbb{R}^+$. To simplify the analysis further, we initially restrict attention to the homogeneous case, for which the cell population evolves in a uniform state. Consequently average $\bar{D}$ in Eqn. (S.6) is replaced by D.

B is treated as an input parameter throughout the Notch-only analysis, enabling us to exploit linearity within equations (S.15) and (S.16) to create a four-equation system for N, H_1, P and D. Application of a steady state assumption ultimately yields the following implicit expression for the steady state concentration of Hes1, $H_1 = H^*$:

$$H^* = \Psi_W\Big(\theta_2\Phi_2(\eta\Phi_1 \circ \Phi_4 \circ \Theta_3(H^*)) + \theta_7\Phi_7(B)\Big)\Theta_2(H^*)\,. \tag{S.26}$$

Since Φ_i is an increasing and Θ_i a decreasing function for $H^* \in \mathbb{R}^+,\forall i$, the composition $\Phi_2(\eta\Phi_1 \circ \Phi_4 \circ \Theta_3(H^*))$ is decreasing in $\mathbb{R}^+$. The right-hand side of (S.26) is therefore a decreasing, positive-valued function of H^* on $\mathbb{R}^+$, because $\Phi(x), \Theta(x) > 0$ for $x > 0$. Equality with the left-hand side, which is trivially increasing in H^*, guarantees the existence of a unique biologically realistic steady state for Hes1.

We now perform linear stability analysis of the steady-state solutions. Eigenvalue analysis of the four-component system suggests the existence of two negative reals, and a pair of complex conjugates with negative real part, as shown in Fig. S.1. Negativity of the real parts of all four eigenvalues generates stable steady states in the system. The complex conjugate eigenvalues arise from a supercritical Hopf bifurcation in θ_2, visible on the lower-right plot of Fig. S.1. That this occurs for $\theta_2 = 0$ suggests that the action of β-catenin upon the Hes1 promoter serves to stabilise Notch and dampen oscillations. Model simulations over a wide range of initial conditions (data not shown) indicate that at lower levels of B, Hes1 steady states increase with the amount of Notch-mediated transcriptional control (i.e. high θ_2 values). Sufficiently high β-catenin expression forces Hes1 into a lower steady state and can dominate Notch-mediated promotion of Hes1.

Analysis of Wnt submodel

Our decoupled Wnt system can be described by Eqns. (S.21) – (S.25), representing the network depicted in Fig. 2A of the main text. The concentration of NICD (F), which interacts with β-catenin, is treated as a model parameter and a constant Wnt stimulus, W, is assumed. An implicit equation for the steady state $B^* = B^*(W, F)$ of β-catenin is as follows:

$$\frac{\alpha_4}{B^*} = \frac{1}{1+W}\left(1 + \frac{\alpha_1}{\nu_F}F\right) + \frac{1.4\,\alpha_2\alpha_3\alpha_5}{\nu_C}\frac{1}{1+(1+W)^4}\,\Phi_6(B^*)^2. \tag{S.27}$$

Since the left-hand side of Eqn. (S.27) is decreasing in B^* and the right-hand side is increasing in B^*, we deduce that there is a unique biologically realistic (i.e. in $\mathbb{R}^+$)

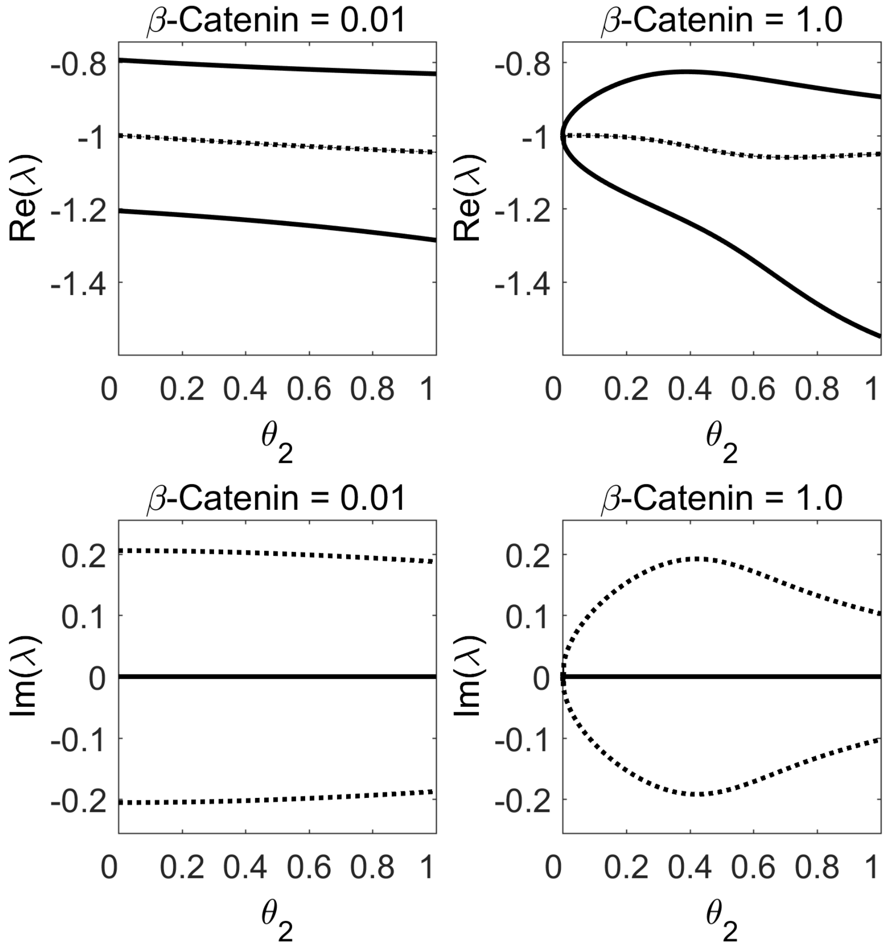

Figure S.1. Eigenvalue dependence in the decoupled Notch system
Dependence of the eigenvalues of the four-component (N, H_1, P, D) Notch system upon θ_2, calculated using Eqn. (S.26). Plots depict (upper plots) real and (lower plots) imaginary components. Complex conjugate eigenvalues are depicted with dashed lines and we have $\nu_H = \nu_P = \nu_D = 1.0$; $\Psi_W = 1.0$; $\eta = 0.5$. Parameter values: $m_i, n_i = 3$; $\kappa_i = 0.5$ for $i = 1, 2, 4$; $\sigma_2 = 0.5$; $\sigma_3 = 0.1$.

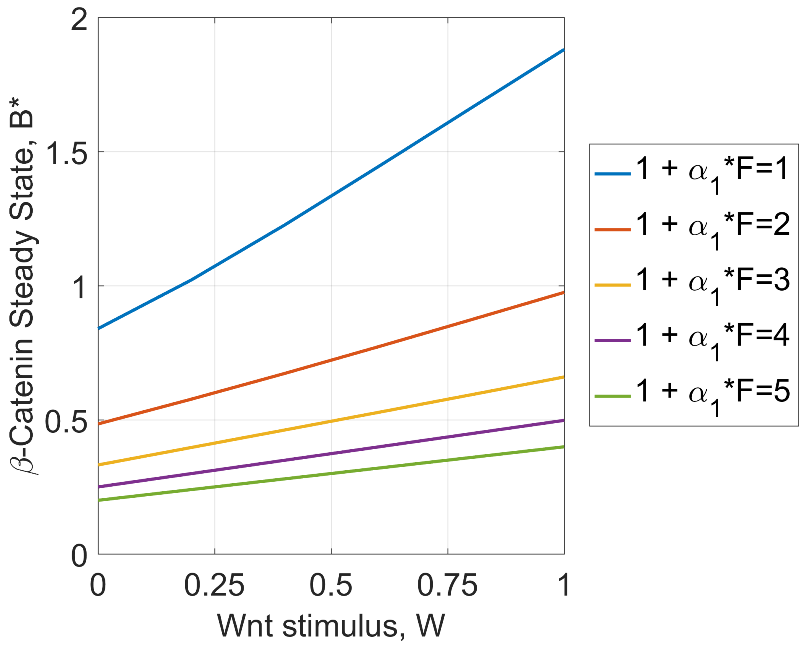

Figure S.2. β-catenin steady state response in the decoupled Wnt system Response of the β-catenin steady state, B^*, described implicitly in Eqn. (S.27), to a variation in Wnt signal W and strength of the Notch interaction, $\alpha_1 F$. Numerical solutions of (S.27) were generated from parameter values $\alpha_2 = \alpha_3 = \alpha_4 = 1.0$, $\alpha_5 = 0.4$ and $\nu_F = \nu_C = 1.0$. In our model, the stronger the interaction with the Notch system (determined by $\alpha_1 F$), the shallower the gradient of B^* and hence the weaker the response of the Wnt system to variation in the extracellular Wnt stimulus.

solution for B^*. Inspection of Eqn. (S.27) confirms that B^* exhibits qualitatively appropriate behaviour within the Wnt system. For example, the steady state value B^* will be increased by:

- increasing the Wnt stimulus, $W(\tau)$;
- increasing α_4, the production rate of β-catenin;
- decreasing α_5, the rate constant for formation of the destruction complex, C;
- decreasing α_3, the rate at which C binds with β-catenin ;
- decreasing the interaction of β-catenin with the Notch system (involving elements of the group $\frac{\alpha_1}{\nu_C} F$).

Strong interaction with NICD attenuates the response of β-catenin to variation in Wnt levels, as shown in Fig. S.2. As the quantity $\alpha_1 F$ increases, the gradient of B^* with respect to Wnt stimulus tends to zero. When the crosstalk with Notch is reduced, B^* increases with the Wnt signal.

A Formal Framework of CRNT

The following definitions and examples aim to provide a brief introduction to the concordance property. A full treatment can be found in [21, 22]. Except where stated otherwise, our definitions and propositions are adopted from Shinar and Feinberg [21].

A biochemical network consists of two aspects: a *network structure* $\{\mathcal{S}, \mathcal{C}, \mathcal{R}\}$, where:

- $\mathcal{S}$ is the set of all *chemical species* in the network, $\mathcal{S} = \{X_1, X_2, \ldots, X_n\}$;

- $\mathcal{C}$ is the set of *complexes*. In CRNT terminology, $\mathcal{C}$ comprises reactant or product expressions which express the stoichiometric linking of elements of $\mathcal{S}$, such as $F + B$, N, and $B + C$ – i.e., any expression which forms the entire right- or left-hand side of a reaction equation;

- $\mathcal{R} \subset \mathcal{C} \times \mathcal{C}$ is the set of *reactions*. Reactions satisfy two properties: first, that $(y, y) \notin \mathcal{R}$ for any $y \in \mathcal{C}$; secondly, that for each $y \in \mathcal{C}$, there exists $y' \in \mathcal{C}$ such that $(y, y') \in \mathcal{R}$ or $(y', y) \in \mathcal{R}$;

and a *kinetics*, $\mathcal{K}$, which assigns a functional form to each of the reaction rates associated with elements of $\mathcal{R}$.

Fig. S.3 demonstrates four example networks: (A) concordant and weakly reversible, (B) concordant and not weakly reversible, (C) discordant and weakly reversible, and (D) discordant and not weakly reversible.

Conventions and Definitions We begin by establishing formal definitions for the *influence specification*, $\mathcal{I}$, and *stoichiometric subspace*, $\mathcal{E}$, of a reaction network, and for the properties of *injectivity* and *concordance*. All definitions adopt the following notation conventions:

- $\mathbb{R}^I$, where I is a set, denotes the vector space of real-valued functions with domain I. This removes the restriction of enumerating vector entries as in $\mathbb{R}^N$ (for $N \in \mathbb{N}$); instead vector entries are indexed over the elements of I;

- Those vector functions which take only positive values form the subset $\mathbb{R}^I_+ \subset \mathbb{R}^I$; those which take non-negative values are indicated by the set $\overline{\mathbb{R}}^I_+ \subset \mathbb{R}^I$;

- The *support* of $\underline{x} \in \mathbb{R}^I$, $supp(\underline{x})$, is the set of indices $i \in I$ for which $x_i \neq 0$;

- For $y \in \mathbb{R}$, $sgn(y)$ denotes the sign of y; for $y \in \mathbb{R}^I$, $sgn(\underline{y})$ indicates the function such that $(sgn(\underline{y}))_i := sgn(y_i)\,, \forall i \in I$.

Having established these notation conventions, we may proceed to some formal definitions. Key definitions are demonstrated for simple networks in Fig. S.3.

Definition 1. *An influence specification $\mathcal{I}$ for a reaction network $\{\mathcal{S}, \mathcal{C}, \mathcal{R}\}$ is an assignment to each reaction $y \to y'$ of a function $\mathcal{I}_{y \to y'} : \mathcal{S} \to \{1, 0, -1\}$ such that*

- $\mathcal{I}_{y \to y'}(s) = 1, \quad \forall s \in supp(y)$,

- *if $\mathcal{I}_{y \to y'}(s) = 1$ [resp. -1], then species s is an inducer [inhibitor] of reaction $y \to y'$;*

- *if $\mathcal{I}_{y \to y'}(s) = 0$, then species s has no influence on the rate of the reaction $y \to y'$.*

The *influence specification* therefore assigns each species a 1, 0 or -1 for every reaction in a network, according to whether the species is an inducer, neutral or an inhibitor of a given reaction. This construct permits the study of networks involving autoregulatory components and allows reactions to be modulated by species other than their products or reactants.

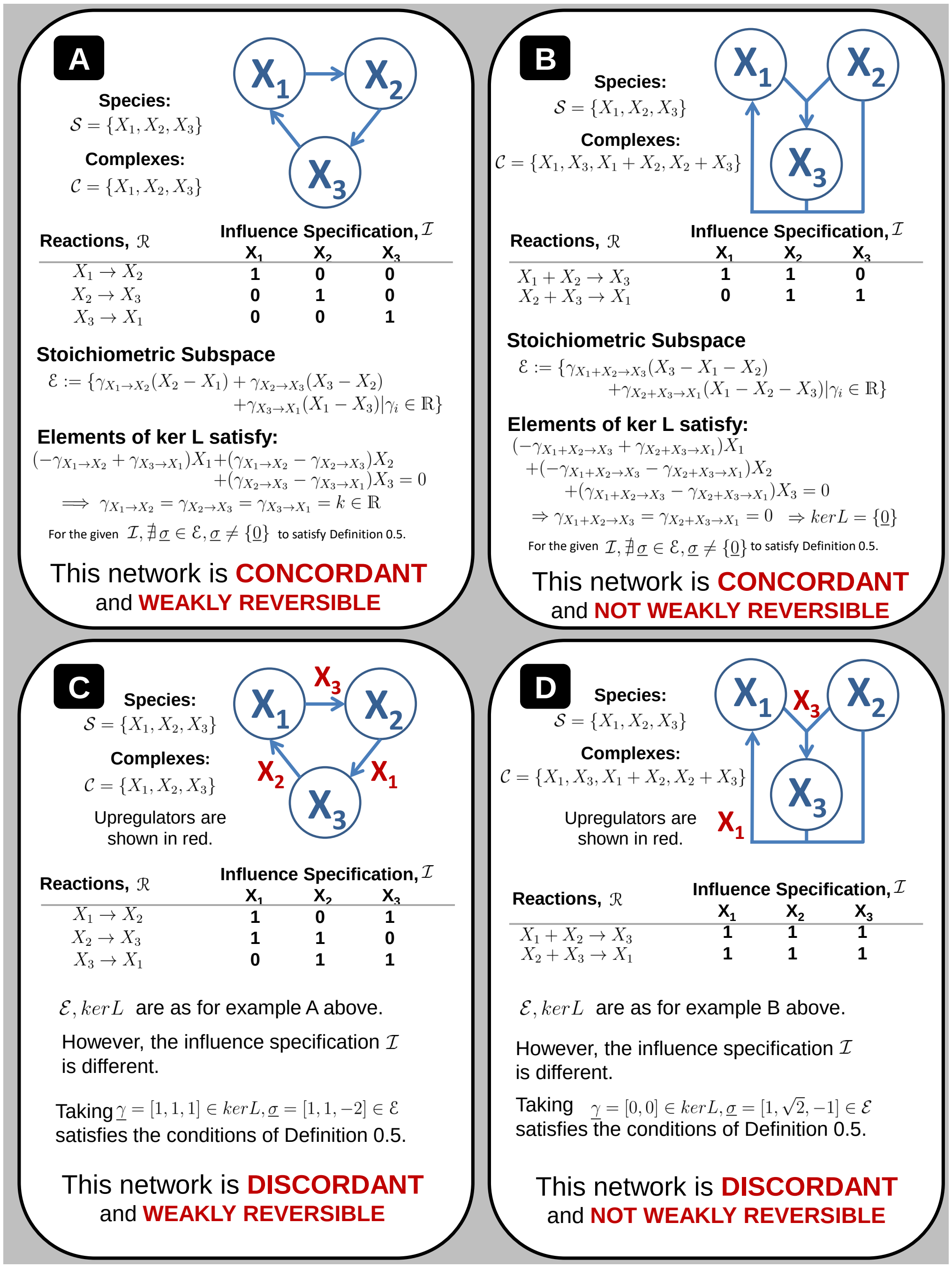

Figure S.3. Simple examples of CRNT network analysis, indicating the sets $\mathcal{S}, \mathcal{C}, \mathcal{R}, \mathcal{I}$ and $\mathcal{E}$. In pairwise cases (A,C) and (B,D), it is the change of influence specification, where new upregulators are introduced, which changes the concordant networks to discordant ones.

Definition 2. *The stoichiometric subspace* $\mathcal{E}$ *of a reaction network* $\{\mathcal{S}, \mathcal{C}, \mathcal{R}\}$ *is the linear subspace of* $\mathbb{R}^{\mathcal{S}}$ *defined by*

$$\mathcal{E} := span\{y' - y \in \mathbb{R}^{\mathcal{S}} | \, y \to y' \in \mathcal{R}\}.$$

Two vectors $\underline{c}, \underline{c}^* \in \overline{\mathbb{R}}^{\mathcal{S}}_{+}$ *are said to be stoichiometrically compatible if* $\underline{c}^* - \underline{c} \in \mathcal{E}$*.*

It is clear from Definition 2 that elements of $\mathcal{E}$ are summations of the linear expressions which arise from rearranging reaction expressions to the form $(products - reactants)$. We also define a linear map $L : \mathbb{R}^{\mathcal{R}} \to \mathcal{E}$ by

$$L\underline{\gamma} := \sum_{y \to y' \in \mathcal{R}} \gamma_{y \to y'} (y' - y) \, , \tag{S.28}$$

for $\gamma_{y \to y'} \in \mathbb{R}$ the entries of $\underline{\gamma}$. The map L is used in Definition 5 to outline concordance. The kernel of L is the set $kerL = \{\underline{x} \in \mathbb{R}^{\mathcal{R}} : L(\underline{x}) = \underline{0}\}$.

All elements of $\mathcal{E}$ can be expressed in the form (S.28). However, the standard approach is to write $\underline{\sigma} \in \mathcal{E}$ as a vector in $\mathbb{R}^{\mathcal{S}}$.

Definition 3. *A kinetics* $\mathcal{K}$ *for a reaction network* $\{\mathcal{S}, \mathcal{C}, \mathcal{R}\}$ *is weakly monotonic with respect to influence specification* $\mathcal{I}$ *if, for every pair of elements* $\underline{c}^*, \underline{c}^{**} \in \overline{\mathbb{R}}^{\mathcal{S}}_{+}$*, the following implications hold for each reaction* $y \to y' \in \mathcal{R}$ *such that* $supp(y) \subset supp(\underline{c}^*)$ *and* $supp(y) \subset supp(\underline{c}^{**})$*:*

- $\mathcal{K}_{y \to y'}(\underline{c}^{**}) > \mathcal{K}_{y \to y'}(\underline{c}^*) \implies \exists$ *species s such that* $sgn(c^{**}_s - c^*_s) = \mathcal{I}_{y \to y'}(s) \neq 0$,
- $\mathcal{K}_{y \to y'}(\underline{c}^{**}) = \mathcal{K}_{y \to y'}(\underline{c}^*) \implies$ *either:*
  (a) $c^{**}_s = c^*_s \quad \forall s \in supp(y)$*, or:*
  (b) $\exists$ *species* s, s' *with* $sgn(c^{**}_s - c^*_s) = \mathcal{I}_{y \to y'}(s) \neq 0$ *and* $sgn(c^{**}_{s'} - c^*_{s'}) = -\mathcal{I}_{y \to y'}(s') \neq 0$.

Weakly monotonic kinetics therefore admit - amongst others - Hill kinetics, mass-action kinetics and hyperbola functions. Our Notch-Wnt ODE model satisfies the conditions for weakly monotonic kinetics subject to its influence specification $\mathcal{I}$.

Definition 4. *A kinetic system* $\mathcal{K}$ *is injective if, for each pair of distinct, stoichiometrically compatible elements* $\underline{c}^*, \underline{c}^{**} \in \overline{\mathbb{R}}^{\mathcal{S}}_{+}$*, at least one of which is positive,*

$$\sum_{y \to y' \in \mathcal{R}} \mathcal{K}_{y \to y'}(\underline{c}^{**})(y' - y) \neq \sum_{y \to y' \in \mathcal{R}} \mathcal{K}_{y \to y'}(\underline{c}^{*})(y' - y).$$

Remark 1. *An injective kinetic system cannot admit two distinct, stoichiometrically compatible equilibria, at least one of which is positive. That is, injectivity may be equated with at most one positive equilibrium.*

The following definition of concordance relies upon the linear mapping L, detailed in Eqn. (S.28).

Definition 5. *A reaction network* $\{\mathcal{S}, \mathcal{C}, \mathcal{R}\}$ *with stoichiometric subspace* $\mathcal{E}$ *is concordant with respect to influence specification* $\mathcal{I}$ *if there do not exist* $\underline{\gamma} \in kerL$ *and a non-zero* $\underline{\sigma} \in \mathcal{E}$ *having the following properties:*

- *For each* $y \to y'$ *such that* $\gamma_{y \to y'} > 0$*, there exists a species* s *for which* $sgn(\sigma_s) = \mathcal{I}_{y \to y'}(s) \neq 0$*;*
- *For each* $y \to y'$ *such that* $\gamma_{y \to y'} < 0$*, there exists a species* s *for which* $sgn(\sigma_s) = -\mathcal{I}_{y \to y'}(s) \neq 0$*;*
- *For each* $y \to y'$ *such that* $\gamma_{y \to y'} = 0$*, either:*
  (a) $\sigma_s = 0 \quad \forall s \in supp(y)$*, or:*
  (b) $\exists$ *species* s, s' *for which* $sgn(\sigma_s) = \mathcal{I}_{y \to y'}(s) \neq 0$ *and* $sgn(\sigma'_s) = -\mathcal{I}_{y \to y'}(s') \neq 0$*;*

Fig. S.3 depicts four simple networks and expressions for $\mathcal{E}$ and $kerL$. The examples in Figs. S.3B and S.3D have trivial kernels; differing influence specifications mean that the first is concordant according to Definition 5, while the other is discordant. Furthermore, for the network in Fig. S.3C, a non-zero $\underline{\sigma} \in S$ and $\underline{\gamma} \in kerL$ can be found which satisfy the conditions listed in Definition 5; this is not the case for Fig. S.3A. The networks shown in Figs. S.3C and S.3D are therefore discordant. None of the examples need be allied with specific reaction rates during this analysis; concordance is a property of the underlying network $\{\mathcal{S}, \mathcal{C}, \mathcal{R}\}$ and is independent of the kinetics $\mathcal{K}$.

Our definitions now established, we turn to the main theoretical result of interest.

Concordant Networks with Weakly Monotonic Kinetics The following proposition provides us with a means of determining when our Notch and Wnt networks (or indeed the full coupled system) are monostable.

Proposition 1. *A kinetic system* $\{\mathcal{S}, \mathcal{C}, \mathcal{R}, \mathcal{K}\}$ *is injective whenever there exists an influence specification* $\mathcal{I}$ *such that:*

- *The kinetics* $\mathcal{K}$ *is weakly monotonic with respect to* $\mathcal{I}$*;*
- *The underlying network* $\{\mathcal{S}, \mathcal{C}, \mathcal{R}\}$ *is concordant with respect to* $\mathcal{I}$*.*

Our Notch-Wnt system has a valid influence specification according to Definition 1 and satisfies Definition 3 of weakly monotonic kinetics. Proposition 1 and Remark 1 together imply that any concordant network or sub-network in our model should be injective and hence monostable. Conversely, a discordant network or sub-network will exhibit more than one steady state and may exhibit nontrivial dynamics.

Parametrisation Details

The following details supplement the description in *Methods and Models - Parametrisation* of the main text.

The Notch model of Shepherd [1] comprises the following equations:

$$\frac{dN}{dt} = -\mu_N N + \frac{\bar{D}^3}{0.5^3 + \bar{D}^3}, \tag{S.29}$$

$$\frac{dH_1}{dt} = -\mu_{H_1} H_1 + \frac{N^3}{0.5^3 + N^3} \times \frac{0.5^3}{0.5^3 + {H_1}^3}, \tag{S.30}$$

$$\frac{dP}{dt} = -\mu_P P + \frac{0.1^3}{0.1^3 + {H_1}^3}, \tag{S.31}$$

$$\frac{dD}{dt} = -\mu_D D + \frac{P^3}{0.5^3 + P^3}. \tag{S.32}$$

The Hernández *et al.* timecourse of Fig. 7 of the main text is characterised by a transient accumulation phase for β-catenin in the two hours post Wnt stimulation, followed by a plateau phase of approximately four hours. During this time, the concentration of the destruction complex remains approximately constant.

We generate initial estimates of α_3 and α_4 as follows. From the data in Fig. 7 of the main text we estimate $B \approx 9$ and $\frac{dB}{dt} \approx 0.5$ at $t \sim 0$, and $B \approx 54$ and $\frac{dB}{dt} \approx 0.0$ at $t \sim 400$. If we substitute these values in Eqn. (S.11), assuming further that $\alpha_1 F + \mu_B \approx 0.007$ and fixing $W = 1.0$, then we obtain simultaneous equations for α_3, α_4:

$$\frac{dB}{dt} = (1 + W)\alpha_4 - B(\alpha_1 \cdot F + \alpha_3 \cdot C + \mu_B),$$

$$0 = 2\alpha_4 - 54(40\alpha_3 + 0.007), \tag{S.33}$$

$$0.5 = 2\alpha_4 - 9(40\alpha_3 + 0.007). \tag{S.34}$$

In deriving estimates for α_3, α_4 from the experimental data of Hernández *et al.* [9], we interpret their Wnt concentration as the reference state and set this to be $W = 1$ in our model. The substitution $\alpha_1 F + \mu_B \approx 0.007 min^{-1}$ is based upon typical values from the decoupled Notch system at steady state. The steady state concentration of C is estimated using data from Tan *et al.* [10]. The solution of Eqns. (S.33) and (S.34) yields initial estimates of $\alpha_3 = 1.028 \times 10^{-4}$ and $\alpha_4 = 0.3$ for use in parameter fitting.

Mean squared error values for the Wnt parametrisation are shown in Table S.8.

References

1. Shepherd S. Computational modelling of neurogenesis; 2010. Unpublished research, University of Nottingham.

2. Momiji H, Monk NA. Oscillatory Notch-pathway activity in a delay model of neuronal differentiation. Phys Rev E: Stat, Nonlinear, Soft Matter Phys. 2009;80(2):021930(13).

3. Collier JR, Monk NAM, Maini PK, Lewis JH. Pattern formation by lateral inhibition with feedback: a mathematical model of Delta-Notch intercellular signalling. J Theor Biol. 1996;183(4):429–446.

4. Peignon G, Durand A, Cacheux W, Ayrault O, Terris B, Laurent-Puig P, et al. Complex interplay between β-catenin signalling and Notch effectors in intestinal tumorigenesis. Gut. 2011;60(2):166–176.

5. Bray SJ. Notch signalling: a simple pathway becomes complex. Nat Rev Mol Cell Biol. 2006;7(9):678–689.

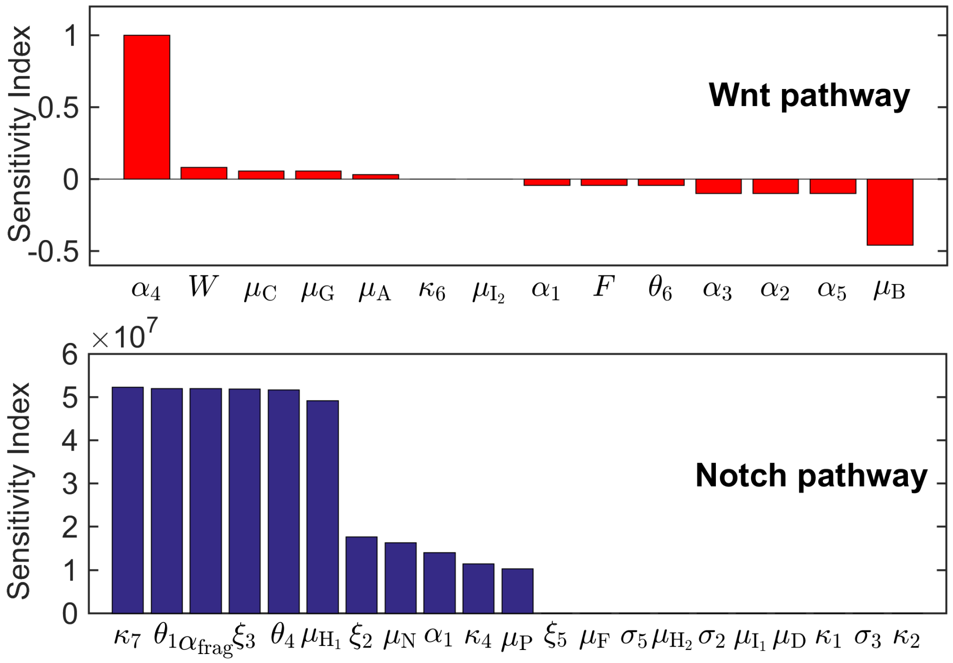

Figure S.4. Results from a preliminary sensitivity analysis for (Top) the steady state of β-catenin in the Wnt system, (Bottom) the oscillation period of Hes1 in the Notch system, showing normalised sensitivities for a 100% increase in the value of each parameter in turn. Values for W and F are included here as a comparison, as they are held constant within the decoupled Wnt system; however they are not varied during parameter fitting, as they are variables rather than parameters in the full system. $x1$ is the multiplier for the Wnt response function $\Psi_{W,A}$, described in the Wnt Pathway Submodel of the SI; α_1 is fitted using the Notch-only system and is not varied during parameter fitting for the Wnt system.

6. Lee E, Salic A, Kruger R, Heinrich R, Kirschner MW. The roles of APC and Axin derived from experimental and theoretical analysis of the Wnt pathway. PLoS Biol. 2003;1(1):116–132.

7. Cho KH, Baek S, Sung MH. Wnt pathway mutations selected by optimal β-catenin signaling for tumorigenesis. FEBS Lett. 2006;580(15):3665–3670.

8. Wawra C, Kühl M, Kestler HA. Extended analyses of the Wnt/β-catenin pathway: robustness and oscillatory behaviour. FEBS Lett. 2007;581(21):4043–4048.

9. Hernández AR, Klein AM, Kirschner MW. Kinetic responses of β-catenin specify the sites of Wnt control. Science. 2012;338(6112):1337–1340.

10. Tan CW, Gardiner BS, Hirokawa Y, Layton MJ, Smith DW, Burgess AW. Wnt signalling pathway parameters for mammalian cells. PLoS One. 2012;7(2):e31882.

11. Mazzoni SM, Fearon ER. AXIN1 and AXIN2 variants in gastrointestinal cancers. Cancer letters. 2014;355(1):1–8.

12. Logeat F, Bessia C, Brou C, LeBail O, Jarriault S, Seidah NG, et al. The Notch1 receptor is cleaved constitutively by a furin-like convertase. Proc Natl Acad Sci U S A. 1998;95(14):8108–8112.

13. Foltz DR, Santiago MC, Berechid BE, Nye JS. Glycogen synthase kinase-3β modulates Notch signaling and stability. Curr Biol. 2002;12(12):1006–1011.

14. Hirata H, Yoshiura S, Ohtsuka T, Bessho Y, Harada T, Yoshikawa K, et al. Oscillatory expression of the bHLH factor Hes1 regulated by a negative feedback loop. Sci Signaling. 2002;298(5594):840–843.

15. Roark R, Itzhaki L, Philpott A. Complex regulation controls Neurogenin3 proteolysis. Biol Open. 2012;1(12):1264–1272.

16. Cole A, Frame S, Cohen P. Further evidence that the tyrosine phosphorylation of glycogen synthase kinase-3 (GSK3) in mammalian cells is an autophosphorylation event. Biochem J. 2004;377:249–255.

17. Yamamoto H, Kishida S, Kishida M, Ikeda S, Takada S, Kikuchi A. Phosphorylation of Axin, a Wnt signal negative regulator, by glycogen synthase kinase-3β regulates its stability. J Biol Chem. 1999;274(16):10681–10684.

18. Agrawal S, Archer C, Schaffer DV. Computational models of the Notch network elucidate mechanisms of context-dependent signaling. PLoS Comput Biol. 2009;5(5):e1000390.

19. Cinquin O. Repressor dimerization in the zebrafish somitogenesis clock. PLoS Comput Biol. 2007;3(2):e32.

20. Collu GM, Hidalgo-Sastre A, Acar A, Bayston L, Gildea C, Leverentz MK, et al. Dishevelled limits Notch signalling through inhibition of CSL. Sci Signaling. 2012;139(23):4405–4415.

21. Shinar G, Feinberg M. Concordant chemical reaction networks. Math Biosci. 2012;240(2):92–113.

22. Shinar G, Feinberg M. Concordant chemical reaction networks and the species-reaction graph. Math Biosci. 2013;241(1):1–23.

	Parameter	Units	Scaling
Dissociation Constants	κ_1	nM	$\kappa_1 = \frac{\theta_4}{\mu_D}\kappa_1'$
	κ_2	nM	$\kappa_2 = \frac{\theta_1}{\mu_{I_1}}\kappa_2'$
	κ_4	nM	$\kappa_4 = \frac{\xi_3}{\mu_P}\kappa_4'$
	κ_6	nM	$\kappa_6 = \frac{\theta_1}{\mu_N}\kappa_6'$
	κ_7	nM	$\kappa_7 = \frac{\theta_1}{\mu_N}\kappa_7'$
Inhibition Constants	σ_2	nM	$\sigma_2 = \frac{\xi_2}{\mu_{H_1}}\sigma_2'$
	σ_3	nM	$\sigma_3 = \frac{\xi_2}{\mu_{H_1}}\sigma_3'$
	σ_5	nM	$\sigma_5 = \frac{\xi_2}{\mu_{H_1}}\sigma_5'$
	σ_K	dim'less	-
Decay Rates	General μ_X	min^{-1}	$\mu_X = \nu_X \mu_N$
Decay Ratios	General $\nu_X = \frac{\mu_X}{\mu_N}$	dim'less	-
Maximal Values	θ_i, $(i = 1, 4, 6)$	nM min^{-1}	-
	θ_i, $(i = 2, 7)$	dim'less	-
	ξ_i, $(i = 2, 3, 5)$	nM min^{-1}	-
Other Constants	α_1	$\text{nM}^{-1}\text{min}^{-1}$	$\alpha_1 = \frac{\mu_N^2}{\theta_1}\alpha_1'$
	α_2	nM min^{-1}	$\alpha_2 = \theta_1\alpha_2'$
	α_3	nM^{-1} min^{-1}	$\alpha_3 = \frac{\mu_N^2}{\theta_1}\alpha_3'$
	α_4	nM min^{-1}	$\alpha_4 = \theta_1\alpha_4'$
	α_5	nM^{-1} min^{-1}	$\alpha_5 = \frac{\mu_G\mu_A}{\theta_6}\alpha_5'$
	α_{frag}	dim'less	-
Exponents	m_i, $i = 1, 2, 4, 6, 7$	dim'less	-
	n_i, $i = 2, 3, 5$	dim'less	-

Table S.2. Parameters associated with the coupled Notch-Wnt ODE model presented in Eqns. (S.1) – (S.13)

**Parameter**	**Half-Life (min)**	**Source**	**Estimated Value (min^{-1})**
μ_N	40.0	Logeat *et al.* [12], human CRC cells	0.017
μ_F	180.0	Foltz *et al.* [13], murine fibroblasts	0.00385
μ_{I_1}	-	*PF*	0.04
μ_{H_1}	22.3	Hirata *et al.* [14], murine myoblasts	0.065
μ_P	14.4	Roark *et al.* [15], *Xenopus laevis*	0.035
μ_D	-	Collier *et al.* [3], Logeat *et al.* [12]	0.049 ($\mu_D = \mu_N$ [3, 12])
μ_{H_2}	-	*PF*	0.0311 (Based upon μ_{H_1} from [14])
μ_G	900.0	Cole *et al.* [16], $HEK293$ cells	9.36×10^{-4}
μ_C	-	Lee *et al.* [6], *Xenopus* oocytes	1.061
μ_B	104.0	Hernandez *et al.* [9], RKO cells	0.00636
μ_A	480.0	Yamamoto *et al.* [17], COS cells	6.23×10^{-4}
μ_{I_2}	-	Lee *et al.* [6], *Xenopus* oocytes	4.204

Table S.3. Dimensional estimates for the decay rate constants of Eqns. (S.1) – (S.13). In the absence of suitable data, our estimates of μ_{H_2} and μ_D have been estimated using other known decay rates. Such approximations are based on factors such as the molecular size of a species and its location and function within the cell. *PF* indicates numerically fitted parameters.

Parameter	Units	Dimensional Value	Source
θ_1	nM min^{-1}	0.06	Agrawal *et al.* [18]
θ_2	dim'less	0.75	Agrawal *et al.* [18], Cinquin *et al.* [19]
θ_4	nM min^{-1}	0.4	*PF*
θ_6	nM min^{-1}	1.64×10^{-4}	Lee *et al.* [6]
θ_7	dim'less	0.25	Agrawal *et al.* [18], Cinquin *et al.* [19] $(1 - \theta_2)$
κ_1	nM	0.6	*PF*
κ_2	nM	0.9	*PF*
κ_4	nM	14.7	*PF*
κ_6	nM	0.026	*PF*
κ_7	nM	10.0	*PF*
$m_i, i = 1, 2, 4, 6, 7$	dim'less	3	-

Table S.4. Dimensional parameters associated with the Hill functions used in Eqns. (S.1) – (S.13). The θ_i are maximal terms; the κ_i are the Hill coefficients; and the m_i are the Hill exponents. *PF* indicates numerically fitted parameters.

Parameter	Units	Dimensional Value	Source
ξ_2	nM min^{-1}	0.5	Agrawal *et al.* [18]
ξ_3	nM min^{-1}	0.9	*PF*
ξ_5	nM min^{-1}	0.9	*PF*
σ_2	nM	3.5	*PF*
σ_3	nM	1.21	*PF*
σ_5	nM	1.7	*PF*
$n_i, (i = 2, 3, 5)$	dim'less	3	Shepherd [1]

Table S.5. Dimensional parameters for the hyperbola functions used in Eqns. (S.1) – (S.13). The ξ_i are maximal rates; the σ_i are constants of inhibition; and the n_i are the exponents. *PF* indicates numerically fitted parameters.

Parameter	Units	Dimensional Value	Source
α_1	nM^{-1} min^{-1}	6.8	*PF*
α_2	nM min^{-1}	0.0174	*PF*
α_3	nM^{-1} min^{-1}	1.465×10^{-4}	*PF*
α_4	nM min^{-1}	0.472	*PF*
α_5	nM^{-1} min^{-1}	0.1044	*PF*
σ_K	dim'less	1.0	Collu *et al.* [20]
α_{frag}	dim'less	0.8	*PF*

Table S.6. Miscellaneous rate parameters used in Eqns. (S.1) – (S.13). *PF* indicates numerically fitted parameters.

Variable	Description	Two-cell ICs (nM)
N	Membrane-bound Notch receptor	0.5
F	Notch Intracellular Domain (NICD)	0.5
H_1	Hes1	0.5
P	Ngn3	0.5
D	Delta ligand	0.5
H_2	Hath1	0.5
G	GSK3β	30.0
C	Destruction complex	25.0
B	Active β-catenin	9.0
A	Axin	27.0
I_1	Intermediate 1 (NICD/β-catenin)	0.5
I_2	Intermediate 2 (GSK3β/β-catenin)	30.0

Table S.7. Initial conditions (ICs) for the variables in the dimensional Notch-Wnt ODE model.

Time (min)	MSE
0	0
30	4.0448
60	0.4595
120	0.3192
240	0.0328
360	0.0029
Total	4.8501

Table S.8. Values for the mean squared error for the final Wnt parametrisation.
